# Household level multidimensional energy poverty and its determinants: Evidence from tea estates in Bangladesh

**DOI:** 10.1371/journal.pone.0354309

**Published:** 2026-08-07

**Authors:** Subrata Koiry, Mohammad Jahangir Alam, Ismat Ara Begum, Md. Shaikh Farid

**Affiliations:** 1 Department of Agricultural Finance and Banking, Sylhet Agricultural University, Sylhet, Bangladesh; 2 Department of Agribusiness and Marketing, Bangladesh Agricultural University, Mymensingh, Bangladesh; 3 Department of Agricultural Economics, Bangladesh Agricultural University, Mymensingh, Bangladesh; 4 Department of Agricultural Marketing and Business Management, Sylhet Agricultural University, Sylhet, Bangladesh; Omeo Kumar Das Institute of Social Change and Development, INDIA

## Abstract

Access to reliable energy is crucial for sustainable livelihood improvements across various dimensions, fostering economic growth. Therefore, addressing energy poverty becomes imperative. This paper aims to estimate the extent of multidimensional energy poverty and its determinants in the tea estates of the Moulvibazar district, Bangladesh. Primary data was collected from 382 tea workers at the Chatlapore tea estate using a multistage random sampling technique. The Multidimensional Energy Poverty Index (MEPI) and the Tobit regression were employed for data analysis. Results indicate a 51% multidimensional energy poverty rate in the study area, with cooking as the most significant contributor. The Tobit model reveals that social and economic factors significantly influence multidimensional energy poverty. Education, occupation, gender, family size, land ownership, access to improved housing, and household earnings are critical determinants. Enhancing household socio-economic status emerges as a key solution to address multidimensional energy poverty. Policies targeting housing improvement, subsidizing and expanding education, and awareness rising on clean energy access, can effectively mitigate multidimensional energy poverty effectively.

## 1. Introduction

Energy is a critical driver of economic growth, with projections indicating a substantial investment of approximately 2.8 trillion US dollars in energy production in 2023 [1]. Despite this investment, around 760 million people worldwide lack access to basic energy amenities, particularly in developing countries [2]. Ensuring universal access to affordable, sustainable, and reliable energy is essential for humankind. In recent years, increased energy demand coupled with rapid population growth has left many households in developing countries without adequate access to basic energy services [3]. Developed nations typically ensure access to modern, clean energy to combat climate change impact and economic prosperity. However, poorer countries like Bangladesh continue to face significant challenges in energy access, leading to energy poverty among their populations.

Energy poverty is commonly defined as the lack of sufficient, modern and reliable energy services [4], which has far-reaching consequences such as health issues, educational barriers, gender disparities [5, 6], air pollution, and food and water scarcity [7], all contributing to perpetuating poverty. Even in affluent nations, energy poverty carries serious societal, health, environmental, and economic implications [8–13]. Given the multidimensional nature of its impact [14], effective public policies are crucial for its eradication. Recognizing the multidimensional impacts of energy poverty underscores the need to address it comprehensively rather than through a single-dimensional lens. Tailored policies are essential as socio-economic conditions vary widely across regions. Therefore, understanding how socioeconomic factors influence energy poverty is pivotal for designing effective policies. In conclusion, addressing energy poverty requires identifying vulnerable groups, understanding the dimensions and causes of their poverty, and tailoring policies accordingly.

With the above background, this paper aims to measure multidimensional energy poverty and its socio-economic causes in the tea estates of Moulvibazar district, Bangladesh. The country is particularly susceptible to multidimensional energy poverty due to the highly inconsistent availability of energy amenities, as evidenced by an energy poverty rate of 36.33% in 2016 [15]. The inability to meet energy demands poses significant challenges, especially in rural areas, where women and children often spend their days searching for cooking fuel, wood for lighting and heating, and other necessities. This situation hampers opportunities for education and human development, contributing to poverty.

Tea estates in Bangladesh are predominantly located in rural areas, and tea workers constitute one of the most marginalized communities in the country. The household-level multidimensional poverty rate among tea workers is reported to be 43% [16]. Earning only 170 Bangladeshi Taka per day (approximately 1.57 US dollars at an exchange rate of 1 US dollar = 108 Bangladeshi Taka) tea workers fall below the World Bank’s poverty threshold (2.15 US dollars per day). This income must cover daily essentials as well as energy costs such as electricity, coal, charcoal, fuel, and gas for lighting, cooking, and heating. Unidimensional energy poverty may indicate a household is energy poor in one aspect but does not necessarily reflect multidimensional energy poverty. For instance, while some tea workers may access electricity or subsidized solar energy for lighting, others may lack such amenities entirely or for specific needs like cooking and heating. Similarly, the availability of kerosene or firewood may vary among households, affecting their energy security differently.

This article addresses several key research questions. Firstly, it examines the extent of household-level multidimensional energy poverty in tea estates. While unidimensional measures like expenditure-based energy poverty exist, a multidimensional approach provides deeper insights into various dimensions of energy poverty. Secondly, it explores which dimensions are most critical for households experiencing energy poverty. Not all households are energy-poor across every dimension, and identifying specific vulnerabilities can inform targeted interventions. Lastly, the study investigates factors influencing energy poverty, considering the unique socio-economic context of tea households compared to the broader population of Bangladesh. In conclusion, examining multidimensional energy poverty in a community already experiencing unidimensional income-based poverty sheds light on complex socio-economic challenges and opportunities for improvement.

However, existing literature has explored energy poverty and its interrelations with various socio-environmental and economic factors. For example, [7] investigated how property ownership, wealth, social, economic, and territorial characteristics impact energy hardship. Similarly, research on socio-economic determinants of energy poverty in African countries has been documented [17–19]. Given the distinct socio-economic status of South Asian countries like Bangladesh compared to African nations, and this study’s relevance is underscored. Few studies have linked energy poverty and temperature variations [20] or explored age as a driver of energy poverty in urban households [21]. While several studies have examined the long-term sustainability, reliability, and cost-effectiveness of energy solutions in rural Bangladesh [22, 23] as well as the adverse impacts of energy poverty on literacy and health [15], few efforts have focused on identifying the specific variables influencing energy poverty in Bangladesh, particularly within the context of tea estates. Thus, this study aims to fill this gap by examining energy poverty and its drivers in these specific settings. Enhanced energy sufficiency can markedly improve living standards by facilitating access to electricity for educational purposes and promoting health benefits through the use of LPG instead of firewood [24–26]. Additionally, it enhances overall quality and efficiency [27].

This research adopts a case-based analytical technique by concentrating on a single tea estate. Although reliance on one estate may restrict statistical generalizability to all tea estates in Bangladesh, it extensively examines household-level energy deprivation within a marginalized and policy-relevant community, and the findings remain still informative. Tea estates in Bangladesh administered under similar wage framework, institutional arrangements, housing facilities, patterns of energy access, and access to fundamental infrastructure. The findings therefore, provide context-specific insights into the mechanisms through which socioeconomic characteristics shape multidimensional energy poverty. These insights are meaningful for understanding energy deprivation in comparable tea labour communities in South Asia and other developing regions.

The objectives of this study are to estimate multidimensional energy poverty by assessing the frequency and severity of such poverty and to evaluate various socio-economic variables to understand the determinants of energy poverty at the household level. This study significantly contributes to the literature by estimating multidimensional energy poverty and identifying its dimensions, potentially marking the first assessment of energy poverty rates in tea estates. Furthermore, based on empirical findings, this paper proposes effective policy implications for alleviating multidimensional energy poverty.

This paper has significant contribution as it has modified the multidimensional poverty index (MPI) to form multidimensional energy poverty index (MEPI) and identify distinct indicators and dimensions on a unique socio-economic context. Thus, it also helps in making a significant policy contribution to improve conditions within the tea community and ensure energy adequacy. The remainder of this paper is organized as follows: Section 2 provides background information on the population. Section 3 summarizes the literature review. Section 4 discusses the data sources, variables, empirical methods used for data analysis. The empirical findings and their discussion are represented in Section 5. Finally, Section 6 concludes the paper and discusses key policy implications.

## 2. Background of the population

Tea labours in Bangladesh are one of the most historically marginalized communities. Most are descendants of workers brought to tea estates during the colonial period and continue to reside within estate settlements that are geographically and socially isolated from surrounding communities. Their livelihoods are highly dependent on tea estate employment, resulting a long lasting labour-management relationship that restricts occupational mobility and other employment opportunities. In spite of several wage revisions over the time, tea labourers continue to receive relatively low wages compared to national living costs and often experience several forms of deprivation regarding to housing quality, sanitation, education, healthcare, and access to modern energy services. For example, Koiry et al. [16] have characterized tea-worker households as a backward community facing continuous multidimensional poverty and social exclusion.

The study area was the Chatlapore tea estate under Lungla valley of the Moulvibazar district in Bangladesh. People who work in tea estates are known as “tea workers,” or “cha sramik,” in local terms. Their duties encompass a wide range of tasks including clearing and preparing soil for tea plantations, cultivating tea plants, weeding, irrigation, fertilization, applying plant protection substances, pruning and shaping tea plants, harvesting tea leaves and processing tea in factories. They also participate in building roads, houses, restrooms, and facilities for tea workers. Tea workers are classified into two categories: temporary and permanent. They typically work eight hours a day, six days a week, with one day off. To earn their daily wage of 1.57 US dollars, women tea workers, who mainly harvest tea leaves, must harvest a minimum of 20 kg of tea foliage within the 8 hours’ workday. They receive an additional fee of approximately $0.009-cent per kilogram for any tea leaves harvested above the 20 kg limit. Male workers earn the same wage performing various tasks on the tea estate. Tea workers can work until they reach 60 or 65 years of age, after which they must retire. Some tea estates temporarily allocate small plots of fallow land to workers for subsistence cultivation. However, such arrangements do not confer legal ownership rights, and tea workers generally do not possess ownership claims over tea-estate land. Those workers who do not get land from estate authority usually receives food support in the form of rice or wheat. Larger households with fewer permanent tea workers also receive rations, even if they own agricultural land. Children of tea workers receive free basic education provided by the tea estate authorities. Temporary tea workers have the freedom to seek work outside of the tea estate but do not receive sick pay or food provisions from the estate. Tea estates may allocate land for housing development, but the workers do not own this land and cannot sell it. Although tea labour constitutes the primary occupation for nearly all surveyed households, some workers often involve in supplementary income-generating activities such as seasonal vegetable cultivation, poultry rearing, fish production, crop cultivation or casual wage labour outside normal tea-estate working hours. These activities usually supplement rather than replace tea-estate employment. In terms of energy use, tea households depend on conventional biomass based sources like firewood, crop residues, and dung cake for cooking and heating [15,23]. The findings in Table 2 indicate that only 2% tea households had access to LPG in the study area, implying limited adoption of modern cooking fuels. This scenario may represent affordability constraints, limited availability of LPG supply facilities, and long-established cooking practices within tea-estate communities. As a result, energy deprivation remains a crucial dimension of poverty among tea-worker households. Workers are permitted to collect leftover tea plants and trees from the plantation area for personal use. Tea estates occasionally provide firewood to workers for household use, especially during festivals.

## 3. Literature review

Numerous studies in the literature have explored the concept and multidimensionality of energy poverty [5,28–30]. These studies consistently highlight that households often lack access to sufficient, reliable, and modern energy services, leading to energy poverty across various dimensions. Adequate access to affordable energy plays a crucial role in enhancing education, healthcare, and economic opportunities beyond the household level [31]. Furthermore, the adoption of clean energy solutions is essential for achieving environmental sustainability [32]. Conversely, energy poverty can have detrimental effects on health, the environment, quality of life, and economic development [33]. Studies investigating the relationship between development and energy poverty underscore these negative effects on agriculture, health, the environment, and the economy [34]. Therefore, considering both the benefits and drawbacks of energy poverty, households must have access to diverse energy sources. The current literature provides a foundation for identifying the determinants of energy deprivation at the household level, aiming to bridge knowledge gaps and ensure equitable access to energy resources for all.

Access to modern energy amenities is increasingly recognized as a critical component of multidimensional energy poverty. Families depriving of dependable electricity and clean cooking fuels often faces adverse outcomes in health, education, economic opportunities, and overall well-being [5,6,23]. In rural and marginalized communities, reliance on biomass fuels exposes household members, especially women, to indoor air pollution and restricts opportunities for educational and socioeconomic progress [5,6,15]. Despite the growing literature on multidimensional energy poverty, evidence concentrating specifically on tea-worker communities remains scarce, particularly in Bangladesh, where households experience continuous socioeconomic deprivation and limited access to modern energy facilities.

However, the literature identifies several factors crucial to understanding energy poverty. Household income and expenditures, income poverty, household characteristics, and deliberate policy interventions are significant contributors to energy poverty [35]. For instance, [36] highlighted the role of financial inclusion in energy poverty in Turkey, while elsewhere emphasize the association between age and energy poverty [36, 37]. Education also plays a vital role, in influencing energy consumption patterns and contributing to environmental conservation [37]. The age of the household head has been shown to affect energy poverty status, with significant implications for energy use, such as gas consumption in the Netherlands [38–40]. Occupational transitions particularly from farming to non-farming occupations have been identified as reducing reliance on traditional fuels like firewood by 14–21% [41]. In contrast to Africa, where studies on multidimensional energy poverty and its determinants are more prevalent [17–19,42], research in Asian countries has primarily focused on measuring multidimensional poverty [21,22,24,43]. Existing studies often concentrate on regional and macro-level assessments of energy poverty. Some have employed demand-based approaches to assess multidimensional energy poverty, exploring its impacts on wellness and literacy in Bangladesh [15,23,44]. Given the diverse socio-economic contexts and mixed effects of determinants, this study aims to fill the knowledge gap by examining how various socio- economic characteristics influence energy poverty and its multidimensional aspects.

This paper contributes novel insights by conducting the first analysis of multidimensional energy poverty and its determinants at the household level in Bangladesh. It uniquely focuses on tea workers, a previously understudied population in terms of energy poverty. This research is pivotal for gaining a deeper understanding of multidimensional energy poverty from a developing country perspective. The findings will be instrumental in informing policy development in Bangladesh aimed at achieving the Sustainable Development Goals (SDGs).

## 4. Methodology

### 4.1. Study area, sampling and data

The Chatlapore tea estate in the Lungla valley of the Moulvibazar district, Bangladesh was chosen as the study area to assess the multidimensional energy poverty. The demographic, infrastructural and institutional characteristics of this study area closely resemble to other tea estates in Bangladesh. For this reason, this study was concentrated in one tea estate. Moreover, this area was chosen because both the conventional and modern energy amenities are found in this area. Also, the location of the study area was far from urban region. Consequently, the spillover effect of urbanization on our rural area has minimized and it helps to measure multidimensional energy poverty more accurately as much as possible. This study employed a cross-sectional survey design with a purely quantitative approach. Primary data were collected from households in the study area using a multistage sampling method. Initially, one valley (i.e., Lungla) out of three in Moulvibazar district was randomly selected. Within Lungla Valley, one tea estate was then chosen randomly. Finally, a total of 384 tea households were selected through simple random sampling technique. A complete list of tea-worker households obtained from the tea-estate authority served as the sampling frame, ensuring that each household had an equal probability of selection. The advantages of simplicity, population representation, applicability to a broader group, and impartiality, among others, make simple random sampling suitable for this study. The specific sample size (n) was determined using Cochran’s formula [45].


$$\begin{array}{c} n=\frac{Z^{\text{2}}p(\text{1}-q)}{d^{\text{2}}} \end{array}$$ (1)

Z represents the standard deviation for a 95% confidence interval, d denotes the margin of error (here, d = 0.05), p is the estimated population proportion (0.50), and q = 1-p. Incorporating these values into the formula yielded an initial sample size calculation of 384 households. Data collection employed a structured questionnaire administered through face-to-face interviews with respondents. The questionnaire was pre-tested in the study area before the execution of final data collection. A total of 384 tea households were initially surveyed. Due to partially missing information (2–3 question was not recorded by the data enumerators for two sampled households), responses from two households were completely excluded, resulting in a final sample size of 382 tea households, which closely aligns with the calculated sample size from Equation 1. Data collection took place between April and August, 2022.

#### 4.1.1. Ethical statement.

Ethical approval for this research was obtained from the Sylhet Agricultural University Research System (SAURES), Sylhet Agricultural University, Bangladesh. The ethics committee endorsed the utilization of verbal informed consent from respondents of the study area due to their lower level of educational background. Prior to each interview, respondents were enlightened about the study objectives, data collection procedures, voluntary participation, and confidentiality safeguards. Verbal consent was obtained from each respondents and documented by the data enumerator in the questionnaire. No identifying information was gathered, and all data were anonymized before analysis to ensure confidentiality.

### 4.2. Variables and description

Before proceeding to the descriptive statistics that depict the socio-economic characteristics of the study area, this study describes the variables listed in Table 2, which illuminate the socio- economic landscape of the study area.

To start with the variable ‘age’, which denotes the age of the household head, is categorized according to the International Labor Organization [46]. Classification: below 15 years (coded as 0), 15–24 years (coded as 1); 25–54 years (coded as 2), and above 55 years (coded as 3). The education is classified into five categories based on Bangladesh`s educational system: illiterate (never goes to school, coded as 0), primary education (up to grade 5, not continuing to grade 6; coded as 1), secondary education (up to grade 10 or equivalent, coded as 2), higher secondary education (up to grade 12, coded as 3); and lastly, tertiary education (bachelor, diploma, masters, and doctoral degrees, encoded as 4). ‘Household size’, is categorized as small (up to 4 members, coded as 1), medium (5–7 members, coded as 2), and large (more than 7 members, coded as 3). In the study area, people usually work in the tea garden to earn a livelihood, but there may also be other families who live in the tea estate but aren’t involved in tea garden work as a primary source of income. ‘Occupation’ includes tea labourers (coded as 0), farmers (primary income from crop production, coded as 1), service sector workers (involved in the job sector for primary earning, coded as 2), business owners (coded as 3), other occupations include casual wage labour outside tea estates, rickshaw pulling, firewood trading, transport-related work, and similar informal income-generating activities(coded as 4). The variable ‘gender’ represents the gender of the household head, categorized as female (coded as 0) or male (coded as 1). The variable ‘number of earning members’ indicates the number of household members earning income from various sources to support household expenses. ‘Total income’ represents the annual earnings of all household members calculated in Bangladeshi Taka (BDT) and converted to US Dollars (USD) at the rate of 1 USD = 108 BDT. ‘Land size’ denotes the area of land owned by a household for agricultural purposes, measured in hectares (ha). ‘Access to electricity, LPG, chimneys, credit and improved housing (such as houses built with tiles, bricks, or cement instead of bamboo, mud, straw, etc.) indicate household access to these facilities or amenities. If households have access to these amenities, they are coded as 1; otherwise, coded as 0 for no access. The energy-related variables in this study are measured as access-based indicators rather than appliance-level usage or electricity service quality measures. Data on household ownership of electrical appliances, electricity consumption intensity, and the duration of power outages was not systematically collected during the household survey. This indicate the contextual characteristics of tea estate communities, where electricity use is limited to fundamental lighting and a small number of low-load appliances, with relatively little variation in appliance ownership across households. Consequently, the lighting dimension includes access to electricity rather than reliability or intensity of use. Yet a small number of surveyed households reported using solar energy for lighting, the majority depended on the traditional electricity network. Information on electricity reliability, including the frequency and duration of load shedding, was not collected. So, the lighting dimension captures access to electricity rather than the continuity or quality of electricity supply.

### 4.3. Multidimensional Energy Poverty Index (MEPI)

This study follows the methodology outlined by Sadath and Acharya [24] to assess the extent and intensity of multidimensional energy poverty. Given the complexity of energy poverty, the study employs numerous indicators to measure and understand it comprehensively. The selection of indicators must be done carefully due to the complexity of the issue. [47] first introduced the multidimensional energy poverty index (MEPI) in the context of energy poverty in African nations. This index was later refined by [48, 49]. The MEPI utilizes an aggregate index to measure energy deprivation, considering both the number of households experiencing energy scarcity and the severity of their energy deficiency. Specifically, the MEPI focuses on fundamental energy needs such as cooking, lighting, and heating or cooling. To quantify these dimensions, various variables are selected based on their relevance and measurability. For instance, lighting availability is assessed by electricity access, while cooking methods are evaluated by the type of fuel used. This study identifies deficiencies related to these fundamental aspects of daily life. Table 1 compiles the dimensions and indicators used to quantify multidimensional energy poverty, including the threshold that determines household disadvantage. Following [24], this study uses three main dimensions—cooking, lighting, and additional measures—to calculate the MEPI. Each dimension is weighted equally at 33% (i.e., 1/3). Similarly, indicators within each dimension are assigned equal weights by dividing the dimension`s weight by the number of indicators. Equal weighting is consistent with the approach of Alkire and Foster [48] and has been used in past studies such as Nussbaumer et al. [47] and Day et al. [50]. The application of equal weights represents both methodological clarity and the unavailability of a strong empirical or normative basis for allocating distinctive significance to particular dimensions. Cooking, lighting, and energy amenities indicate necessary and complementary aspects of household well-being, and emphasizing one dimension over another would introduce subjective value judgments. Therefore, equal weighting permits the poverty index to stay easily understandable, policy-neutral, and comparable to past MEPI based studies. Also, this approach has been extensively adopted in multidimensional poverty measurement when consensus on relative importance among dimensions is lacking. This approach assumes that each dimension contributes equally to overall deprivation and helps in minimizing subjective bias due to assigning unequal weights. Table 1 presents the weights assigned to all indicators and dimensions utilised in this study to compute the MEPI.

**Table 1:** Dimensions and indicators for measuring multidimensional energy poverty.

Dimensions	Indicators	Deprivations	Weight
Cooking	LPG	Household has no access to LPG (liquefied petroleum gas)	1/6
	Type of stove	Household has stove without a chimney	1/6
Lighting		Household has no access to electricity	1/3
Additional measures	Firewood	Household use firewood for cooking, lightening and heating	1/15
	Dung cake	Household use dung cake for cooking, lightening and heating	1/15
	Crop residue	Household use crop residue for cooking, lightening and heating	1/15
	Kerosene	Household use kerosene for cooking, lightening and heating	1/15
	Coal/charcoal	Household use coal or charcoal for cooking, lightening and heating	1/15

Each indicator in the study is assigned a binary score (0 or 1) based on whether it signifies energy poverty or not. For example, a household without electricity is coded 1 indicating energy poverty, while a household with electricity is coded as 0 within the lightning dimension. In many parts of the study area, electricity infrastructure from the government or the estate was absent. So, households had no scope to use electricity. Likewise, LPG supply points were located far from most households, requiring them to spend around 5.55 US dollar (i.e., 600 Bangladesh Taka @ 1 US dollar = 108 Bangladesh Taka) on transportation to purchase LPG. These circumstances indicate that the primary cause of energy deprivation is limited access rather than affordability. Therefore, in the cooking category, a household without access to LPG and using a stove without a chimney (e.g., a conventional stove) is coded as 1 indicating energy poverty otherwise, it is coded as 0. Lastly, a household using kerosene, coal or charcoal, dung cake, firewood, or crop waste for heating, lighting, and cooking is assigned a score of 1 (indicating poverty), otherwise 0. Each component`s weight is then multiplied by its respective score to determine the level of energy poverty. Lastly, the MEPI combines these scores to calculate the overall index. The MEPI captures two parameters: the incidence of energy poverty or headcount ratio (H), which indicates the proportion of the population experiencing energy poverty, and the poverty gap or intensity of energy poverty (A), which measures the average extent of deprivation across indicators. Therefore, the MEPI synthesizes both the incidence and intensity of energy poverty and can be represented as Equation 2;


$$\begin{array}{c} MEPI=H\times A \end{array}$$ (2)

Where MEPI denotes the multidimensional energy poverty index, H represents the headcount ratio, and A represents the intensity of energy poverty.

The incidence of energy poverty was calculated using Equation 3:


$$\begin{array}{c} H=\frac{q}{n} \end{array}$$ (3)

Where q is the number of energy-poor household, n is the total number of households.

The intensity of energy poverty was estimated by using Equation 4 below:


$$\begin{array}{c} A=\sum_{i=\text{1}}^{n}C_{i}(k)q \end{array}$$ (4)

Where q is the number of energy-poor people, n is the total number of households, Ci is the sum of the i^th^ weighted deprivation, and k is the deprivation cut-off.

This study adopts the deprivation cut-off 33.33%, established by Alkire et al. [51], which has been widely used in past studies [47,50]. Given that each of the three dimensions carries equal weight, this cut-off implies that a household is categorized as energy poor if it is deprived in at least one dimension or an equivalent combination of indicators across dimensions. Conceptually, this cut-off minimizes the risk of under-identifying households facing considerable energy deprivation against the risk of overstating poverty due to marginal shortfalls. Moreover, previous studies have shown that MEPI estimates are generally robust to reasonable variations in the cutoff, typically within the 30–40% range [51,52]. Therefore, adopting the 33.33% cut-off ensures both conceptual consistency and comparability with the existing literature.

### 4.4. Tobit regression analysis

To examine the impact of socioeconomic background on energy poverty, a variety of social and economic factors are considered. These factors encompass characteristics such as income, housing type, house size, employment, education, ecological and geographical diversity, among others [9,18,53–56], which may influence energy poverty. Given the diverse factors contributing to multidimensional energy poverty, empirical analysis of the effects of socioeconomic characteristics on households’ multidimensional energy poverty is crucial for informing policy development. For truncated data, this study employs the Tobit model to explore the relationship between non-negative response variables and regressors. The deprivation values C_i_ for multidimensional poverty (truncated in regression) serves as the response variable in this study. C_i_ value ranges from 0 (indicating minimal deprivation, left censored), to 1 (maximum deprivation, right censored), representing absence or presence deprivation across dimensions. Therefore, a dual-bounded Tobit model is utilized to obtain robust regression estimates. The model can be written as Equation 5 and 6;


$$\begin{array}{c} Y_{j}^{*}={\delta X}_{j}+\varepsilon_{j} \end{array}$$ (5)

$$\begin{array}{c} Y_{j}=\left\{ \begin{array}{c} \text{0}\\ \text{1} \end{array}{\;Y}_{j}\right.\;,\begin{array}{c} ifY_{j}^{*}\leq \text{0}\\ \;if\text{0}<Y_{j}^{*}<\text{1}\\ ifY_{j}^{*}\geq \text{1} \end{array} \end{array}$$ (6)

Where Y_j_ is the dependent variable, ranges between 0 and 1 representing a distributive dependent variable constrained within these bounds. The disturbance term ε captures the residual error in the model, j denotes the total number of observations, X indicates the regressors for the j^th^ observations, and δ represents the coefficient vector associated with these regressors.

## 5. Empirical results and discussion

### 5.1. Descriptive statistics

Table 2 also delineates that the age of household heads is 41 years on average, and a significant majority, 77% of family heads fall within the prime working age range of 25–54 years. This suggest that most household heads are within an age range typically associated with high productivity and employment, which can have implications for more economic activity, higher income levels, and accessibility of modern and environment friendly energy amenities. Education levels among tea household heads show that 42% are uneducated, while among the literate, the majority have completed only primary level education, accounting for 30%. The lower level of education might have resulted in ignorance and influence households to access required energy facilities. Yet, tertiary education was found relatively uncommon (2%) in the study area suggesting that a small number of individuals with higher education stayed within tea-estate probably due to of family ties, limited employment opportunities, or engagement in supervisory and administrative activities related with tea-estate operations. Family sizes in the study area are fairly evenly distributed, with 45% of households classified as small, and 47% as medium. Small households may have lower energy demand due to fewer members but they could be at higher risk of energy poverty because of lower combined income, making it harder to afford energy cost. Medium-sized households might have balanced energy consumption pattern in terms of sharing energy cost due to a handful number of family members and pooled higher income. But energy poverty can still be a challenge for medium families if income is not proportional to number of household members. The possibility of energy poverty at medium families can be anticipated since 98% of households engage in tea estate work (i.e., low wage-oriented occupation) as their primary occupation to sustain their livelihoods. This preference likely stems from the customary residence of people in tea estates, making tea labour (or tea production-related work) the prominent choice for employment.

**Table 2:** Descriptive statistics.

Variables	Unit	Mean/Percent
Age		
Below 15 years (0)	%	0
15 to 24 years (1)	%	6
25 to 54 years (2)	%	77
Above 55 years (3)	%	17
Average	Years	41
Education		
Illiterate (0)	%	42
Primary education (1)	%	30
Secondary education (2)	%	23
Higher secondary education (3)	%	3
Tertiary education (4)	%	2
Household size		
Small (1)	%	45
Medium (2)	%	47
Large (3)	%	8
Occupation		
Tea labourers (0)	%	98.69
Farmers (1)	%	0.26
Service sector workers (2)	%	0.52
Business owners (3)	%	0.26
Other occupations (4)	%	0.27
Gender		
Female (0)	%	41
Male (1)	%	59
Access to improved housing		
No access (0)	%	77
Access (1)	%	23
Access to credit		
No access (0)	%	27
Access (1)	%	73
Access to electricity		
No access (0)	%	6
Access (1)	%	94
Access to LPG		
No access (0)	%	98
Access (1)	%	2
Access to chimney		
No access (0)	%	95
Access (1)	%	5
Number of earning members	Person	2
Total income	US dollar	892.70
Land size	Ha	0.21

Source: Author estimation, 2024.

In the study area, the majority of households are headed by males, comprising 59% of the total. Additionally, about 73% of households have accessed credit from lending institutions, and 94% of households have electricity connections. However, only 23% of families reside in developed houses, and a minimal 2% of households can use LPG for cooking. Similarly, only 5% of households use stoves equipped with chimneys. These findings highlight the prevalence of energy poverty in the study area. On average, each household in the study area is supported by 2 earning members, and the average annual income is approximately 893 US dollars. In terms of agricultural production, the average land size per household is 0.21 hectares (ha).

### 5.2. Multidimensional energy poverty

Table 3 present’s estimate of the multidimensional energy poverty rate, incidence of poverty, and its severity among tea workers using Equations (2), (3), and (4). In the study area, the multidimensional energy poverty rate was found to be 51%, which surpasses the national-level estimate of 36% [7]. This suggests that a majority of households in tea estates face multidimensional energy poverty. Comparatively, Bangladesh has shown a decline in its national-level energy poverty rate from 2005 to 2016 [15], indicating a potential further reduction by 2024. However, higher energy poverty rates in tea communities imply greater challenges in accessing energy compared to urban and or other rural areas of the country. The incidence of energy poverty, or headcount ratio, was estimated at 0.96, indicating that 96% of households experience multidimensional energy poor and encounter multiple deprivations. The intensity of poverty (i.e., the poverty gap) was estimated at 0.52, suggesting that energy-poor households lack 52% of the weighted indicators. Comparable studies in the literature show similar findings. For instance, Senegal reported a multidimensional energy poverty rate of 48.7%, while Tongo recorded 78.5% [17]. Ghana showed a 36.3% energy poverty rate [18], whereas China reported 14% in [21], and the Philippines 37% [22]. These comparisons underscore the severity of energy poverty in lower-income countries in Africa, akin to the findings in this study. Despite Bangladesh`s relatively similar national-level energy poverty rate compared to the Philippines and China, the household level energy poverty rate in tea estates is notably higher. This disparity is attributed to the overall poverty in tea communities, supported by a multidimensional poverty rate of 43% [16]. Furthermore, barriers such as affordability and traditional practices hinder the adoption of cleaner energy sources like LPG. Most households in tea estates lack access to improved stoves (95%) or LPG (98%), relying instead on traditional fuels like firewood, crop residues, and dung cake, readily available due to agricultural activities and livestock rearing. In tea estate households’ women typically prepare dung cakes from cow dung in the dry season (i.e., winter), ensuring a year-round supply, especially crucial during the monsoon. Both male and female members, whether employed or not in tea estates, often collect parts of tea plants or large trees for cooking throughout the year and heating during the monsoon and winter seasons, benefiting from these resources at no cost. This reliance on freely available resources discourages them from switching to alternative energy sources.

**Table 3:** Multidimensional energy poverty status of tea workers.

Energy poverty parameters	Value
Incidence of energy poverty (H)	0.96
Intensity of energy poverty (A)	0.52
Multidimensional energy poverty (MEP) rate	0.51
Multidimensional energy non-poor people	0.04

Source: Authors estimation, 2024.

Most tea estates in Bangladesh, including our study area, are located in rural areas and heavily rely on agriculture, wood, and crop residues. Households commonly store crop residues such as straw for heating and cooking purposes. However, these traditional fuels emit heavy smoke, contributing to indoor and outdoor air pollution. Women, who primarily cook for their families in tea estates, bear the brunt of this smoke exposure. The absence of a gas pipeline network in tea estates and urban areas further solidifies the population`s dependence on traditional fuels. Despite electricity being a safer and more eco-friendly option for cooking, cooking and cooling or heating purposes, its adoption among tea communities remains low. This reluctance could stem from higher costs and limited awareness.

Table 4 identifies the most significant dimension contributing to multidimensional energy poverty among tea workers. Among the three dimensions analysed (cooking, lighting, and additional measures), cooking was identified as the primary contributor, accounting for 93% of energy poverty (Table 4). This finding aligns closely with the results reported by [18], who similarly identified cooking as a major component of energy poverty. The high percentage attributed to cooking underscores the prevalent use of biomass fuels among the population in the study area. This highlights the urgent need for community education and awareness campaigns aimed at promoting the transition to modern cooking fuels such as LPG or electricity.

**Table 4:** Contribution of dimensions to multidimensional energy poverty.

Dimensions	Percentage contribution
Cooking	93
Lighting	6
Additional measures	1

Source: Authors’ estimation, 2024.

### 5.3. Determinants of energy poverty

This section delineates the results of the Tobit model used to estimate the determinants of multidimensional energy poverty in tea estates, with findings detailed in Table 5. The analysis regressed poverty scores, derived from weighted indicators using Equation 2, against 10 variables known to influence multidimensional energy poverty using STATA. Among these variables, 6 were used as dummy variables. These are education, occupation, gender, access to credit, access to improved housing, and household size. For education, the illiterate category; for occupation (i.e., primary), the tea estate workers; for gender, females as household heads; for access to credit, tea households who took credit; and for access to improved housing, households who owned houses built with cement, brick, or tiles were designated as the base. The analysis expectedly showed significant impacts, underscoring the influence of these factors on energy poverty in the tea estate communities.

**Table 5:** Determinants of multidimensional energy poverty in tea estates.

Variables	Coefficient	Standard error	t-statistics	P > |t|
**Age**	−0.0002	0.0004	−0.50	0.615
**Education**				
Primary	0.013	0.011	1.04	0.297
Secondary	−0.0007	0.014	−0.05	0.959
Higher secondary	−0.031	0.029	−1.09	0.276
Tertiary	−0.065*	0.039	−1.69	0.092
**Occupation**				
Farming	−0.019	0.097	−0.20	0.842
Service	−0.040	0.068	−0.58	0.560
Business	−0.044	0.098	−0.45	0.650
Other occupation	0.326***	0.097	3.33	0.001
**Gender**				
Male	−0.025**	0.010	−2.31	0.021
**Access to credit**				
Access	0.001	0.011	0.09	0.929
**Access to improved housing**				
Access	−0.028**	0.012	−2.33	0.020
**Household size**				
Medium	0.019*	0.011	1.75	0.081
Large	0.041**	0.021	1.95	0.050
**Land size**	−0.025***	0.009	−2.83	0.005
**Number of earning members**	−0.021**	0.008	−2.49	0.013
**Ln income**	−0.025	0.016	−1.59	0.114
Constant	0.297*	0.178	1.68	0.094
Sigma (σ)	0.096	0.003		
LR (χ^2^)	49.56***			
Prob > χ^2^	0.000			
Number of observations	382			

Source: Authors estimation, 2024.

*** Significant at 1% level, ** Significant at 5% level, *Significant at 10% level.

The results of this study revealed that the coefficient of tertiary education was estimated at – 0.065, which was statistically significant at the 1% level. This suggests that households headed by individuals with tertiary education experience a 6.5% lower incidence of multidimensional energy poverty compared to households headed by illiterate individuals. These results underscore the critical role of education in combating energy poverty by empowering individuals with knowledge and decision-making capabilities to adopt better energy sources and improve household welfare. Previous research has consistently shown that as the educational attainment of the household head increases, the likelihood of multidimensional energy poverty decreases [18,44,57,58]. Although the coefficients for secondary and higher secondary education were not statistically significant, their negative signs indicate that educated household heads are more likely to mitigate energy poverty compared to illiterate heads. The coefficient of occupation was also statistically significant at the 1% significance level, with an estimated value of 0.326. This suggests that households primarily dependent on occupations other than tea labouring (such as rickshaw pulling, or day labouring) experience a 32.6% higher incidence of multidimensional energy poverty compared to household’s dependent on earning from tea labour in the study area. The relatively higher incidence among these households may stem from lower earnings associated with day labour and other occupations (occupations mainly consisted of casual and informal labour activities characterised by unstable earnings and limited access to employment-related benefits.) outside the tea estate, which may not adequately support reducing energy poverty after meeting other basic life expenses such as education, healthcare and food. Conversely, despite lower wages, tea labourers benefit from subsidies provided by the tea estate authorities, including essential provisions like medicine, rationed rice or flour, processed tea, housing and sanitation facilities. In contrast, households primarily engaged in farming, service, or business showed negative coefficients (Table 5), indicating potential earnings higher than those of tea-labour households, although these coefficients were not statistically significant.

Table 5 also presents the estimated coefficient for gender, which was calculated at 0.025, and found to be statistically significant at the 5% level. This indicates that multidimensional energy poverty is 2.5% lower in male-headed households compared to female-headed households, highlighting gender as a significant determinant of multidimensional energy poverty. This finding aligns with previous research indicating higher levels of multidimensional energy poverty in female-headed households compared to male-headed households [18,44]. Female- headed households may face greater challenges in adopting new and developed energy sources, primarily due to cost barriers. Furthermore, the possession of specific household assets such as a stove with a chimney, plays a crucial role in determining the multidimensional energy poverty status. In a multidimensional context, female-headed households are more likely to experience energy poverty due to lower ownership of such assets.

The coefficient for access to improved housing was estimated at −0.028, indicating statistical significance at the 5% level. This suggests that households owning improved housing experience 2.8% lower energy poverty compared to those without such housing. This result is consistent with existing literature, which highlights that features like pakka walls (improved housing) contribute to reducing energy poverty [44]. Households with improved housing often prioritize maintaining their living conditions, including preventing smoke that can damage walls. Consequently, they are more inclined to adopt modern energy services such as electricity, and LPG-driven stoves for cooking. This shift towards cleaner energy sources plays a significant role in alleviating energy poverty.

Household size emerges as a significant determinant of multidimensional energy poverty. The empirical findings reveal that larger families tend to experience higher levels of energy poverty. The estimated coefficients for medium (0.019) and large household sizes (0.041), statistically significant at the 1% and 5% levels, respectively, indicate that multidimensional energy poverty is 1.9% and 4.1% higher in medium and large households compared to small households in the study area. Previous studies also support a positive association between family size and energy poverty [57, 58]. The increased incidence of energy poverty in medium and large households can be attributed to diversified priorities and basic demands, leading to competing needs for limited household resources [59]. Larger households may opt for cheaper yet more harmful domestic fuels to manage costs. Moreover, decision-making processes in larger families may result in unplanned expenditures, whereas smaller families typically manage with fewer priorities and more structured expenses. Additionally, the coefficients for land size and number of earning members were estimated at 0.025 and 0.021 respectively, significant at the 1% and 5% levels. This suggests that an increase of 1 hectare (ha) of cultivated land and an additional earning member in a household decrease the multidimensional poverty rate by 2.5% and 2.1%, respectively. Access to more land for cultivation enables households to generate higher incomes from farming, potentially facilitating investments in safer and modern energy sources. Similarly, higher-earning members contribute more to covering the costs associated with accessing modern energy facilities, thereby aiding in the reduction of multidimensional energy poverty.

In addition, the variables age, access to credit, and income were found to be statistically insignificant. The coefficients for age and income were estimated to be negative, indicating that as age and income increase, energy poverty decreases in households. However, the statistical insignificance of income should not be interpreted as evidence that household earnings are unimportant for reducing energy poverty. Instead, the impact of income may be indirectly captured by correlated socioeconomic factors such as housing quality, land ownership, occupational status, and the number of earning members, all of which represents underlying household welfare. This indicates that income does not exert an additional independent effect once these correlated characteristics are controlled for in the model. Nevertheless, income-generating opportunities and wage improvements remain central to addressing energy deprivation among tea workers. These results also suggest that with age household heads may gain experience and become more focused on sustainable managing their livelihoods and making improvements. This finding is consistent with other research indicating an inverse relationship between age and the adoption of 21st century energy sources [58]. Elderly household heads tend to prefer conventional biomass fuels for cooking [60]. Furthermore, an increase in income may enhance the financial sustainability of tea workers, enabling them to invest in LPG and other modern energy services for cooking, lighting, and other needs. Lastly, the estimated coefficient for access to credit was found to be positive in this study. This suggests that having access to credit may increase liabilities, as part of a household`s income may be allocated to loan repayment, potentially limiting funds available for purchasing LPG or other clean fuels for cooking, lighting, or heating and cooling purposes.

### 5.4. Diagnostic test results

#### 5.4.1. Multicollinearity, heteroskedasticity and normality test.

To ensure the reliability of the estimated Tobit model, several diagnostic tests were conducted. Table 6 presents the findings of the multicollinearity, heteroskedasticity and normality tests using the Variance Inflation Factor (VIF), Breusch–Pagan (BP), and Shapiro–Wilk methods, respectively. An Ordinary Least Square (OLS) regression was estimated before performing the VIF, BP, and Shapiro–Wilk tests.

**Table 6:** Diagnostic test results.

Diagnostic test	Statistics	P value	Interpretation
Multicollinearity (VIF)	Mean VIF = 1.24	–	No multicollinearity issue
Heteroskedasticity (BP)	χ² = 11.11	0.000	Heteroskedasticity present
Normality (Shapiro–Wilk)	W = 0.894	0.112	Normality exists
Robustness (Bootstrap, 1000 reps)	–	–	Coefficients stable Confirms robustness

The findings in Table 6 show that the estimated mean VIF value is 1.24, suggesting the absences of multicollinearity issue. The null hypothesis of the BP test depicts the constant variance, which was rejected, indicating the presence of heteroskedasticity. To deal with this problem and ensure valid inference, the Tobit model was estimated by using robust standard errors, which corrects the standard errors and test statistics for heteroskedasticity. Additionally, a bootstrap analysis with 1,000 replications was conducted, confirming that the coefficient estimates are stable and robust. These steps address potential bias due to heteroskedasticity and support the reliability of our results. This bootstrapping reduced the potential bias due to heteroskedasticity and ensure the reliability of the findings.

The estimated value of Shapiro-Wilk statistic was 0.849, which is closer to 1, indicating normality. Moreover, the estimated p value (0.112) is greater than 0.05, implying a failure to reject the null hypothesis (i.e., residuals are normally distributed).

#### 5.4.2. Estimation of ordinary least square (OLS) regression.

The estimation of ordinary least square (OLS) is presented in Table 7. Table 7 shows similar findings as Table 5, which means that Tobit estimates are consistent. Household size, land size, number of earning members, access to improved housing, gender, occupation, and education were found to be statistically significant, whereas age, access to credit, and income were insignificant statistical determinants of household-level multidimensional energy poverty. The more or less similar outcome of both models assures the importance of statistically significant socio-economic variables in the policy formulation aimed at multidimensional energy poverty eradication for tea workers’ sustainable livelihood progress, especially at the micro or regional level.

**Table 7:** Estimation of ordinary least square (OLS).

Variables	Coefficient	Standard error	t-statistics	P > |t|
Age	−0.0002	0.0004	−0.49	0.623
Education				
(1) Primary	0.013	0.013	1.02	0.309
(2) Secondary	−0.0007	0.014	−0.05	0.960
(3) Higher secondary	−0.031	0.029	−1.06	0.288
(4) Tertiary	−0.065*	0.040	−1.65	0.010
Occupation				
(1) Farming	−0.019	0.100	−0.19	0.846
(2) Service	−0.040	0.070	−0.57	0.569
(3) Business	−0.044	0.101	−0.44	0.658
(4) Other occupation	0.326***	0.100	3.25	0.001
Gender				
(1) Male	−0.025**	0.011	−2.26	0.025
Access to credit				
(1) Access	0.001	0.011	0.09	0.930
Access to improved housing				
(1) Access	−0.028**	0.012	−2.28	0.023
Household size				
(1) Medium	0.019*	0.011	1.71	0.088
(2) Large	0.041**	0.022	1.89	0.059
Land size	−0.025***	0.009	−2.76	0.006
Number of earning members	−0.021**	0.008	−2.43	0.015
Ln income	−0.025	0.016	−1.55	0.112
Constant	0.297*	0.183	1.62	0.105
F	2.97***			
Prob > F	0.000			
Number of observations	382			

Source: Authors’ estimation, 2024.

*** Significant at 1% level, ** Significant at 5% level, *Significant at 10% level

#### 5.4.3. Model specification test.

To check the reliability and ensure the robustness of findings, a model specification test was conducted to check whether the Tobit model was estimated using the right functional form and included the relevant explanatory variables. The test was conducted by using the *linktest* command in STATA 18 and the findings are reported in Table 8. The test included the predicted value of hat (*hat*) and its squared term (*hatsq*) as regressors. The findings shows that the squared term (*hatsq*) was statistically insignificant (p value is 0.915), indicating that the model was correctly specified, no incorrect functional form was used, and no relevant variables were omitted.

**Table 8:** Model specification test.

Variables	Coefficient	Standard Error	t	P > |t|
Hat	0.902	0.431	2.09	0.037
Hatsq	0.089	0.838	0.11	0.915
constant	0.026	0.015	1.73	0.085
LR (chi2)	49.589			
Prob > chi2	0.000			

Source: Authors’ estimation, 2024.

*** Significant at 1% level, ** Significant at 5% level, *Significant at 10% level.

## 6. Conclusion

This paper aims to assess the extent of multidimensional energy poverty in tea estates, focusing on the Chatlapore tea estate in the Moulvibazar district, Bangladesh, as representative of all tea estates. It extensively investigates various socioeconomic factors and their statistically significant associations with household-level multidimensional energy poverty. The findings reveal that approximately 51% of households in tea estates experience multidimensional energy poverty, with cooking identified as the most significant contributing dimension. The regression analysis emphasizes the substantial influence of social and economic characteristics on multidimensional energy poverty. Except for age, access to credit, and income, variables such as education, occupation, gender, family size, land size, access to improved housing, and the number of earning members significantly influence energy poverty. The study underscores the importance of enhancing household socioeconomic conditions to improve access to clean energy and affordable home energy solutions. The perseverance of energy poverty among tea workers should be interpreted within the historical and socioeconomic structure of tea estates in Bangladesh. Tea workers have historically faced social marginalization, limited upward mobility, and restricted livelihood diversification, with sustained dependence on estate-based employment systems. These structural constraints reduce household ability to accept cleaner and more reliable energy sources, even where basic energy infrastructure is available. Consequently, addressing energy poverty in this context requires more improvements in physical access that strengthen social inclusion, labour welfare, income stability, and overall living conditions within tea-growing communities.

Nevertheless, these findings hold crucial policy implications for decision-makers aiming to mitigate multifaceted energy poverty. Family composition and access to improved housing emerge as critical factors influencing energy poverty, with larger households and those in improved housing less susceptible. The study identifies higher energy poverty among female-headed households, attributable to their dual responsibilities of tea leaf plucking and household cooking. To address energy poverty effectively, the government could initiate housing schemes or partnerships with tea estates, where dependence on traditional cooking fuels is high, providing initial subsidies for LPG or establishing rural gas pipeline networks could alleviate energy poverty. Education plays an important role, underscoring the need for programs that promote education and awareness of clean energy technologies among tea workers. Finally, households with more earning members and large land holdings experience lower energy poverty, highlighting the potential of programs that increase household income through livelihood diversification, skill development or increased land-based productivity. Thus, improving housing, education, and clean energy access, especially for female-headed and larger households can effectively reduce multidimensional energy poverty in tea estates.

However, this study has some limitations. Firstly, it is limited to a single tea estate, which may limit the generalization of findings. But the institutional and socioeconomic similarity of tea estates in Bangladesh indicates that the observed relationships are likely to hold in similar tea-labour based communities, making the findings analytically transferable rather than statistically representative. Secondly, the analysis depends on access-based indicators of energy amenities and does not incorporate appliance-level electricity usage or electricity reliability measures such as the frequency and duration of power outages. These aspects can affect household well-being and effective energy use but were beyond the scope of the present household survey. Consequently, the lighting dimension mainly represents electricity availability rather than service quality or utilization intensity. Furthermore, while a small number of households reported access to solar energy, alternative electricity technologies were uncommon in the study area. Therefore, future research can strengthen the evaluation of multidimensional energy poverty by incorporating electricity reliability indicators, involving load shedding frequency, duration, role of alternative energy sources such as solar home systems, information on appliance ownership, electricity usage patterns, and supply reliability to provide a more comprehensive measure of functional energy access. Future research may also include employment contract type (permanent versus temporary workers), access to forest resources, rationing services provided by tea estates, and participation in government social safety net programmes. These institutional and welfare-related factors may affect household energy choices and energy deprivation but were beyond the scope of the present study. Despite these limitations, this study provides a robust baseline assessment of energy poverty in tea estate communities, where cooking-related energy poverty remains the dominant challenge. The outcomes bear significant implications for designing effective measures to combat energy poverty at micro, regional, and national levels.

## Supporting information

S1 File Questionnaire. (PDF)

S2 File MEP Dataset. (XLSX)
