# Supplementary material for: Household level multidimensional energy poverty and its determinants: Evidence from tea estates in Bangladesh
Source: PLoS One. 2026 Aug 7;21(8):e0354309. doi: 10.1371/journal.pone.0354309 (PMC13450794; doi:10.1371/journal.pone.0354309)
Supplement: Supplementary file 1 [file pone.0354309.s001.pdf]

DEPARTMENT OF AGRICULTURAL FINANCE AND BANKING
SYLHET AGRICULTURAL UNIVERSITY, SYLHET-3100

<u>Interview Schedule on Tea Workers</u>

Household-level Multidimensional Energy Poverty and Its Determinants: Evidence from Tea Estates in Bangladesh

You are invited to participate in a research study titled "Household-level Multidimensional Energy Poverty and Its Determinants: Evidence from Tea Estates in Bangladesh." The purpose of this study is to understand the energy conditions of households in tea estates and the factors influencing energy access and use.

Your participation is voluntary. You may choose not to answer any question that makes you uncomfortable, and you are free to withdraw from the survey at any time without any negative consequences.

The information you provide will be used only for academic research purposes. All responses will be kept strictly confidential and will not be shared with anyone outside the research team. Your identity will not be disclosed in any report, publication, or presentation resulting from this study. Data will be presented in aggregate form so that no individual respondent can be identified.

This survey does not involve any physical, financial, or legal risk. However, some questions may require you to recall personal household conditions. If you feel discomfort, you may skip such questions.

By agreeing to participate, you acknowledge that you have understood the purpose of the study and your rights as a participant. If you have any questions about the research, you may contact the researcher.

<u>Consent:</u>

Do you agree to voluntarily participate in this study?

Yes ☐

No ☐

Sample No. ☐ **Mobile No:**

1. Information of the Respondent:

(i) Name: ……………………… (ii) Estate: ……………….

(iii) Occupation: Main*:…………Subs……….. (iv) Age: (years)…………

[Tea worker = 1, casual = 2, Farmer =3, Service =4, Business =5, others= 6]

(v) Gender of Household Head : 1.Male……… 2. Female ……. 3. Others…..

(vi) Education and age of family members:

Family members	Age	Education	Working status
Father:			
Mother:			
Child 1:			
Child 2:			
Child 3:			
Child 4:			
Child 5:			
Other:			
Other:			
Other:			

(vii) Floor of the living room/house : ……… [dirt/mud/sand/dung = 1, cement = 2 tiles=3]

(viii) cooking fuel:…… [Firewood = 1, crop residue = 2, cow dung = 3, LPG = 4, Electricity =5]

(ix) type of stove: …….. [mud = 1, gas = 2, electric = 3] chimney :………… [y =1, n=0]

(x) Have electricity? ………………………..(Yes =1, No = 0)

(xi) Have solar ? …………. (Yes =1, No = 0) Self…………….. From Govt………

(xii). Fuel for lighting: ………… [kerosene = 1, firewood = 2, Electricity = 3, others =4]

(xiii) Do you use dung cake for heating/cooking/lighting?......................... [Yes = 1, No = 0]

(xiv) Do you use firewood for heating/cooking/lighting?........................... [Yes = 1, No = 0]

(xv) Do you use crop residue for heating/cooking/lighting?....................... [Yes = 1, No = 0]

(xvi) Do you use kerosene for heating/cooking/lighting?........................... [Yes = 1, No = 0]

(xvii) Do you use coal/charcoal for heating/cooking/lighting?.................... [Yes = 1, No = 0]

(xviii) Did you take credit:……… [Yes =1, No = 0] Source:…………………………………

(xix) Number of earning members in family………………………………….

(xx) Total own cultivated crop land (decimal): ………………………………

(xxi) Do you always cultivate your own land (Yes =1, No=0) : …………………..

(xxii) Land status:

Lease in	Lease out	Lease value	Rent in	Rent out	Rent value	No of times in year
Taka						
Crop						

2. Wage status of tea workers:

Weak	No. of worker	Total wage	original salary per person	Sick payment (day/taka)	Ration	Fund deduction	Electricity charge
1							
2							
3							
4							

3. Other household income:

Enterprise/Source	Area/Number	Quantity produced	Price	Total
Crops				
Fruits/vegetables				
Tree/wood				
Shrimp/Fish				
**Livestock**				
Cattle				
Goat				
Milk				
Poultry/duck				
Eggs				
Wage				
Salaries/month				
Business/trading/month				
Remittances/year				

Others				
Agricultural Equipment				
Total				

5. In the absence of electricity what you use for lighting?..............

[nothing = 0, kerosene = 1, Wood = 2, others = 3]

6. When you use cow dung for cooking? …………… [all the year round = 0, monsoon = 1]

Signature of surveyor with Date:

Thank you for your participation in this study. Your responses will provide important insights into the nature and determinants of household-level multidimensional energy poverty in the tea estates of Bangladesh. All information you have provided will remain confidential and will be used solely for academic research purposes.

If you have any questions regarding this study, please feel free to contact the researcher at following address.

Subrata Koiry
Assistant Professor
Department of Agricultural Finance and Banking
Sylhet Agricultural University, Sylhet-3100
Contact: +880 1738441393
Email: skoiry.afb@sau.ac.bd
