# Supplementary material for: Household level multidimensional energy poverty and its determinants: Evidence from tea estates in Bangladesh
Source: PLoS One. 2026 Aug 7;21(8):e0354309. doi: 10.1371/journal.pone.0354309 (PMC13450794; doi:10.1371/journal.pone.0354309)
Supplement: Supplementary file 3 [file pone.0354309.s005.docx]

Overall Comments:

The authors portray a useful research work centering on the energy poverty of the tea workers and their families living in a ghettoized situation with restricted opportunities for social mobility and bare minimum basic services provided by tea estates or tea garden owners who treat the tea workers as their bonded laborers. A study on the tea estates in Bangladesh that captures the plights of tea workers and their family members is significant from different viewpoints. Therefore, the authors’ attempt to reflect on their struggle to manage energy poverty provides a timely contribution to the knowledge world. However, like their unconventional poverty due to their ghetto-like conditions and bonded labor-like status, their energy poverty deserves an unconventional study covering contextual dimensions of poverty. Their poverty stems from extreme level of manipulation of labor ownership and deprivation of minimum living wages and basic needs. Therefore, income poverty, resource poverty and energy poverty among the tea workers should have been navigated through a more flexible framework that allows the researchers to capture more nuanced and intersectional dimensions of poverty, which may not necessarily resemble to the experiences of different other marginalized communities in Bangladesh.

Specific comments:

1. The background section does not provide detailed information about tea workers’ unique kind of poverty and plights stemmed from their ghettoized situation and bonded labor-owner relationships that restrict their social mobility and extreme level of extraction of labor resources in exchange for a sarcastic level of unfair wages and deprivation of basic needs.
2. The background section includes some information that needs to be verified. For example, the information about land ownership provisions for tea workers should be supported by authentic sources if there are any such provisions. Ideally, tea workers are not supposed to own any land in a tea estate. However, sometimes, tea garden authorities lease out some fellow lands to the workers for cultivating crops. This does not mean any ideas of land ownership that the tea workers are allowed in tea gardens. Please refer to the sources of information if there is any.
3. This is also misleading that tea workers have engagement in commercial activities and even in agriculture, fish cultivation, poultry, cow and goat rearing etc. that should be counted as alternative livelihood options. The occupation-wise sample distribution of this study does not also support this idea. Therefore, please refer to authentic sources of this information.
4. The intro and background do not provide sufficient reflection on the sources of energy. Even, literature review does not reflect anything on tea workers’ access to energy and their multidimensional poverty.
5. The methodology does not detail it out if the samples were selected randomly from a list of workers. Or the selection process just followed the ideas of selecting households through a systematic random sampling process.
6. Why an open-ended questionnaire has been used is not clarified, when a multidimensional poverty framework clearly indicates a question structure and response options.
7. Contract types, i.e., permanent or temporary workers, could have been an important dependent variable as the workers’ income mostly depends on how they are contracted for their work in tea gardens. Moreover, access to forest resources for cooking fuel and heating room during winter could have been another variable. Again, access to rationing services provided by tea garden owners or other social safety net supports provided by government could have been another variable.
8. The energy poverty dimensions should have included not only accessing electricity but also the level of having uninterrupted services given the reality of managing the supply through loadshedding that mostly affects the rural areas. In that situation, what are the alternative sources for electricity could have been included. Is it solar panel or is there any other alternative?
9. In occupation-wise analysis, does it make sense to compare with other types of occupations, when the tea workers are 98.69%? Rather, the categories like full-time workers, part-time workers, full-time workers plus other income sources, part-time workers and other income sources could have made more sense. Table 5 and 7 underline this point as the occupation like farming, service and business are found to be not statistically significant in determining energy poverty in tea gardens. However, other occupations have been found to be significant, though, it has not been defined what it means by other occupations.
10. Are there any tea workers who have tertiary education and still work in tea garden and remain in poverty?
11. Why income does not have significant implication in determining energy poverty should be revisited, because improved housing, number of earning members, occupations are closely linked with income. This finding may give a wrong message to the policymakers and tea garden owners regarding the long, undisputed and unfair low wages in the tea gardens of Bangladesh.
12. This study underlines the importance of improving socioeconomic conditions of tea workers to help decrease their energy poverty but does not reflect on the issues of other practical dimensions of tea workers’ poverty that are associated with their subjugation, bonded labor and ghetto situation.
13. This paper requires a thorough revision to address these observations.
