# Supplementary material for: Household level multidimensional energy poverty and its determinants: Evidence from tea estates in Bangladesh
Source: PLoS One. 2026 Aug 7;21(8):e0354309. doi: 10.1371/journal.pone.0354309 (PMC13450794; doi:10.1371/journal.pone.0354309)
Supplement: Supplementary file 4 [file pone.0354309.s004.docx]

**Response letter to reviewer#1**

Journal: PLOS ONE

Title: Household Level Multidimensional Energy Poverty and Its Determinants: Evidence from Tea Estates in Bangladesh

ID: PONE-D-25-43666

Dear reviewer #1,

Thank you so much for reviewing our manuscript and offer us with positive and detailed comments. We prepare following responses to explain what we have done in the revision. In order to help reviewers and editor to follow what we have revised and changed, we keep all changes tracked in the revised version, and mark responses in this response letter with color ‘blue’ and double quotation marks (“…”). All the black words are originally from review#1 that we cite with double citation mark and explanation of logic in response to comments but not changed in reviewed manuscript.

**Major comments:**

“Overall comment: This paper deals with measuring energy poverty and its determinants in tea estates in Bangladesh. The study is based on primary data collected for the specific study. The paper may be informative in the context of Bangladesh. However, the quality of paper needs to be improved for larger global audience.”

Response to comment 1:

Thank you very much for your positive feedback and suggestions to improve the paper.

“1. Since the data was collected by the authors, I feel that some of the important aspects could have been collected carefully. For e.g. whether energy poverty is due to access or affordability. If there is no reliable supply of LPG, it is natural that deprivation is due to access and not due to affordability alone. The authors may know the ground situation of the study area and incorporate the same in the paper.”

Response to comment 1:

We thank the reviewer for this observation. In the study area, LPG supply points are located far from most households, requiring respondents to spend around 600 Bangladesh Taka for transportation to purchase LPG. This indicates that the primary cause of energy deprivation is limited access rather than affordability alone. We have clarified this in the revised manuscript, highlighting that physical distance to LPG outlets constrains usage, while the associated transportation cost adds a secondary financial burden. Our changes are documented (**see line number: - number 298-303**) in the revised manuscript (track change mode).

“In many parts of the study area, electricity infrastructure from the government or the estate was absent. So, households had no scope to use electricity. Likewise, LPG supply points were located far from most households, requiring them to spend around 5.55 US dollar (i.e., 600 Bangladesh Taka @ 1 US dollar = 108 Bangladesh Taka) on transportation to purchase LPG. These circumstances indicate that the primary cause of energy deprivation is limited access rather than affordability.”

“2. Authors could have collected information on other variables like using household appliances using electricity to strengthen the measurement of electricity access. Further, information on its reliability could have been collected. These variables could be added to the lighting index as well. If this information is available, authors may incorporate the same.”

Response to comment 2:

We thank the reviewer for the valuable suggestion. Unfortunately, our survey did not collect detailed information on household appliances or electricity reliability. We mainly focused on the availability of basic facilities. We acknowledge that including such variables could enhance the measurement of electricity access and the robustness of the lighting index. We have clearly noted this as a limitation in the revised manuscript (**lines: - 619-623**). We have also discussed the potential implications of this limitation for interpreting our results and suggested it as an avenue for future research.

“However, this study could not incorporate detailed information on household appliances due to respondents’ reluctance. This limitation may result in an incomplete evaluation of electricity access, as the lighting index mainly focuses on availability rather than usage. Future research could incorporate appliances ownership and reliability indicators to deliver a comprehensive assessment of electricity access.”

“3. Check the age code in page 10 for those with 25 to 54 years.”

Response to comment 3:

We also noticed missing codes for some variables and we added that in the revised manuscript. There were code mistakes according to your comment. Thank you for scrutinize comment. We corrected as follows (**See lines 244-245; page 11 and lines: - 266-267, page 12 for the adding description of codes).**

“25 to 54 years (coded as 2), and above 55 years (coded as 3). If households have access to these amenities, they are coded as 1; otherwise, coded as 0 for no access.”

“4. Why don’t you mention the variables in Table 2 instead of the codes used in the model. For example, age categories are mentioned instead of actual age group. There is nothing wrong but the reader will have to remember the codes mentioned earlier or frequently go back and forth to check what the category. It will affect the ease of reading the paper. Same thing applied for other categories as well.”

Response to comment 4:

“Thank you for your comment. We have included the actual names instead of code in our revised manuscript-track change mode. (**see table 2).** For ease we are not presenting here the table.

5. Table 2 can be made more informative. For example, instead of just presenting sample’s frequency distribution, why not cross tab it with different dimensions of energy poverty and with the overall energy poverty score. That will give a lot more information than the present one.”

Response to comment 5:

“We appreciate your suggestion regarding Table 2. The primary purpose of Table 2 is to provide a descriptive overview of the sample characteristics, which is standard practice in empirical studies. Cross-tabulating at this stage would conflate descriptive presentation with analytical results, potentially creating redundancy and obscuring clarity.

The relationships between household characteristics and energy poverty dimensions are systematically examined in subsequent tables and regression analyses (**TOBIT**), which provide a more rigorous and interpretable assessment. We think that, presenting cross-tabulations in the main descriptive table might not add substantial interpretative value but could complicate the presentation for readers.

Therefore, we maintain that the current format of Table 2 , which we think is methodologically appropriate and sufficient for conveying sample composition.”

“We hope our response will satisfy you.”

6. Authors could have presented a detailed account of deprivation as seen in Sadath and Acharya (2017). For example, giving a detailed picture of extent of deprivation of the households across deciles or some other measure. What is the need to make sense of insignificant coefficients about the result?”

Response to comment 6:

“We thank you for this observation and insightful comment. However, the objective of the present study was not to provide a descriptive decomposition of deprivation, but rather to empirically identify the key determinants influencing household deprivation. The type of analysis suggested—such as the distribution of deprivation across income deciles, as in Sadath and Acharya (2017)—is valuable for exploratory assessment, but it does not directly contribute to the estimation and interpretation of the determinants of deprivation, which is the central focus of this paper.

Moreover, the manuscript already includes descriptive statistics that summarize the extent and pattern of deprivation among the sampled households. Adding another level of descriptive breakdown might divert attention from its econometric focus.

Regarding the discussion of insignificant coefficients, these were not interpreted as meaningful effects, but were mentioned briefly to demonstrate that certain variables did not exhibit a statistically significant influence once other covariates were controlled for. We think, presenting and acknowledging insignificant results is a standard econometric practice, as it provides a complete picture of the model outcomes and helps to identify which factors are or are not empirically relevant.

For these reasons, we consider the current analytical structure can be appropriate for addressing the stated objectives of the study.”

“7. In line number 496 on page 26 there is ‘15’ number mentioned. Is it citation or something else? If citation, it is necessary to put it in bracket.”

Response to comment 7:

That one is typo error. We have deleted that. You will find the deletion on **line number 514 at page number 27** in our revised manuscript (track change mode). Thank you.

“8. Let the recommendations of the study stem from the findings and not well-intentioned statements. For e.g. work-life balance, equal wage, etc.”

Response to comment 8:

“Thank you very much for these important observations. We have rearranged some lines, deleted some lines and rewritten the recommendation part in our conclusion section. You will find that in the revised manuscript (Track change mode: **lines:- 602-618**). However, we are presenting the change and new part here in below:”

“The study identifies higher energy poverty among female-headed households, attributable to their dual responsibilities of tea leaf plucking and household cooking. To address energy poverty effectively, the government could initiate housing schemes or partnerships with tea estates, where dependence on traditional cooking fuels is high, providing initial subsidies for LPG or establishing rural gas pipeline networks could alleviate energy poverty. Education plays an important role, underscoring the need for programs that promote education and awareness of clean energy technologies among tea workers. Finally, households with more earning members and large land holdings experience lower energy poverty, highlighting the potential of programs that increase household income through livelihood diversification, skill development or increased land-based productivity. Thus, improving housing, education, and clean energy access, especially for female-headed and larger households can effectively reduce multidimensional energy poverty in tea estates.”

**Note that, changing (correcting) recommendation, signaled us to change recommendation in Abstract and we did so, which you will find in the lines between 35-38 in the revised manuscript (Track change).**

”9. Overall, the study examines a socially sensitive issue and redressal of the same has micro, macro and global implications. However, the paper falls short in making a compelling case for itself. I feel there is lot of scope for improvement. Authors may take the findings sportively and improve the paper.”

Response to comment 9:

Thanks a lot for your comment. We have tried to improve our paper according to your feedback. Now, we hope that this paper have dealt enough with its shortcomings.

At the end, Thanks a lot for your valuable feedback that helps to improve this papers’ quality. We expect that you will be satisfied from our response, but, still if you have any opinion, please, you are most warmly welcomed.

**Response letter to reviewer#2**

Journal: PLOS ONE

Title: Household Level Multidimensional Energy Poverty and Its Determinants: Evidence from Tea Estates in Bangladesh

ID: PONE-D-25-43666

Dear reviewer #1,

Thank you so much for reviewing our manuscript and offer us with positive and detailed comments. We prepare following responses to explain what we have done in the revision. In order to help reviewers and editor to follow what we have revised and changed, we keep all changes tracked in the revised version, and mark responses in this response letter with color ‘blue’ and double quotation marks (“…”). All the black words are originally from review#1 that we cite with double citation mark and explanation of logic in response to comments but not changed in reviewed manuscript.

**Major comments:**

“1. Generalizability concerns arise because the sample is drawn solely from the Chatlapore tea estate (see Lines 207–217), yet the title and abstract imply a national scope. This restricts the ability to generalize findings to all tea estates in Bangladesh.”

Response to comment 1:

Thank you very much for this crucial observation. The Chatlapore tea estate was carefully selected because its socioeconomic and infrastructural characteristics closely resemble those of other tea estates in Bangladesh. The estate represents a typical case in terms of worker livelihood patterns, energy access, and institutional arrangements common across the tea sector. Therefore, while the study does not aim to make statistical generalizations to all tea estates, it provides analytical generalization and policy-relevant insights applicable to similar tea estate communities in Bangladesh. Furthermore, the objective of the paper is to understand the multidimensional nature of energy poverty within the tea estate context, not to make national-level estimates. Hence, the study’s scope and conclusions remain consistent with its design and contribution. However, we made the following changes in the lines **209-213 in revised manuscript (Track mode).**

“The Chatlapore tea estate in the Lungla valley of the Moulvibazar district, Bangladesh was chosen as the study area to assess the multidimensional energy poverty. The demographic, infrastructural and institutional characteristics of this study area closely resemble to other tea estates in Bangladesh. For this reason, this study was concentrated in one tea estate. Moreover, this area was chosen because both the conventional and modern energy amenities are found in this area.”

“2. Methodological justification is lacking for several key decisions. The use of equal weights for MEPI dimensions is presented without empirical or theoretical support (Lines 276–279). The 33.33% poverty cutoff is adopted from Alkire et al. without sensitivity analysis or rationale for its appropriateness (Lines 308–309).”

Response to comment 2:

Thank you very much for providing positive review and comment. We have addressed your comment by following way –

““Line **287-290** (in revised manuscript) : Equal weighting is consistent with the approach of Alkire and Foster [48] and has been used in past studies such as Nussbaumer et al. [47] and Day et al. [59]. This approach assumes that each dimensions contributes equally to overall deprivation and remove subjective bias due to unequal weights.”

“**Line 325-330** (in revised manuscript): This study adopts the 33.33% cut-off rate established by Alkire et al. [50], which has been widely used in past studies [47,59] to identify households experiencing deprivation across various dimensions and to determine both the headcount ratio and poverty intensity. Previous research also suggests that findings are generally robust to reasonable variations in the cutoff (e.g., 30–40%) [50, 60]. The choice of this benchmark allows for comparability with existing studies and provides a standard reference for interpreting energy poverty in the study area.””

“3. Robustness and model checks are missing. No alternative specifications or tests for multicollinearity among categorical variables are presented (see description of model variables, Lines 424–431 and Table 5). There are also no diagnostic tests for Tobit assumptions reported in the results section.”

Response to comment 3:

**“**Again, we want express our appreciation for your valuable comments to improve this paper. We have done several diagnostic tests and report them in a separate sub-section (5.4) under section 5. The name of subsection is 5.4. diagnostic test result. We mentioned the changes here in blue color text. The changes lies between lines 535-580.

**“**5.4 Diagnostic test results

*5.4.1 Multicollinearity, Heteroskedasticity and Normality test*

To ensure the reliability of the estimated Tobit model, several diagnostic tests were conducted. Table 6 presents the findings of the multicollinearity, heteroskedasticity and normality tests using the Variance Inflation Factor (VIF), Breusch–Pagan (BP), and Shapiro–Wilk methods, respectively. An Ordinary Least Square (OLS) regression was estimated before performing the VIF, BP, and Shapiro–Wilk tests.

The findings in Table 6 show that the estimated mean VIF value is 1.24, suggesting the absences of multicollinearity issue. The null hypothesis of the BP test depicts the constant variance, which was rejected, indicating the presence of heteroskedasticity. To deal with this problem and ensure valid inference, the Tobit model was estimated by using robust standard errors, which corrects the standard errors and test statistics for heteroskedasticity. Additionally, a bootstrap analysis with 1,000 replications was conducted, confirming that the coefficient estimates are stable and robust. These steps address potential bias due to heteroskedasticity and support the reliability of our results. This bootstrapping reduced the potential bias due to heteroskedasticity and ensure the reliability of the findings.

The estimated value of of Shapiro-Wilk statistic was 0.849, which is closer to 1, indicating normality. Moreover, the estimated p value (0.112) is greater than 0.05, implying a failure to reject the null hypothesis (i.e., residuals are normally distributed).

Table 6: Diagnostic test results

| Diagnostic test | Statistics | P value | Interpretation |
| --- | --- | --- | --- |
| Multicollinearity (VIF) | Mean VIF = 1.24 | - | No multicollinearity issue |
| Heteroskedasticity (BP) | χ² = 11.11 | 0.000 | Heteroskedasticity present |
| Normality (Shapiro–Wilk) | W = 0.894 | 0.112 | Normality exists |
| Robustness (Bootstrap, 1000 reps) | - | - | Coefficients stable  Confirms robustness |

*5.4.2 Estimation of ordinary least square (OLS) regression*

The estimation of ordinary least square (OLS) is presented in Table 7. Table 7 shows similar findings as Table 5, which means that Tobit estimates are consistent. Household size, land size, number of earning members, access to improved housing, gender, occupation, and education were found to be statistically significant, whereas age, access to credit, and income were insignificant statistical determinants of household-level multidimensional energy poverty. The more or less similar outcome of both models assures the importance of statistically significant socio-economic variables in the policy formulation aimed at multidimensional energy poverty eradication for tea workers’ sustainable livelihood progress, especially at the micro or regional level.

Table 7: Estimation of ordinary least square (OLS)

| **Variables** | **Coefficient** | **Standard error** | **t-statistics** | **P > \|t\|** |
| --- | --- | --- | --- | --- |
| Age | -0.0002 | 0.0004 | -0.49 | 0.623 |
| Education |  |  |  |  |
| (1) Primary | 0.013 | 0.013 | 1.02 | 0.309 |
| (2) Secondary | -0.0007 | 0.014 | -0.05 | 0.960 |
| (3) Higher secondary | -0.031 | 0.029 | -1.06 | 0.288 |
| (4) Tertiary | -0.065* | 0.040 | -1.65 | 0.010 |
| Occupation |  |  |  |  |
| (1) Farming | -0.019 | 0.100 | -0.19 | 0.846 |
| (2) Service | -0.040 | 0.070 | -0.57 | 0.569 |
| (3) Business | -0.044 | 0.101 | -0.44 | 0.658 |
| (4) Other occupation | 0.326*** | 0.100 | 3.25 | 0.001 |
| Gender |  |  |  |  |
| (1) Male | -0.025** | 0.011 | -2.26 | 0.025 |
| Access to credit |  |  |  |  |
| (1) Access | 0.001 | 0.011 | 0.09 | 0.930 |
| Access to improved housing |  |  |  |  |
| (1) Access | -0.028** | 0.012 | -2.28 | 0.023 |
| Household size |  |  |  |  |
| (1) Medium | 0.019* | 0.011 | 1.71 | 0.088 |
| (2) Large | 0.041** | 0.022 | 1.89 | 0.059 |
| Land size | -0.025*** | 0.009 | -2.76 | 0.006 |
| Number of earning members | -0.021** | 0.008 | -2.43 | 0.015 |
| Ln income | -0.025 | 0.016 | -1.55 | 0.112 |
| Constant | 0.297* | 0.183 | 1.62 | 0.105 |
| F | 2.97*** |  |  |  |
| Prob ˃ F | 0.000 |  |  |  |
| Number of observations | 382 |  |  |  |

Source: Authors’ estimation, 2024.

*** Significant at 1% level, ** Significant at 5% level, *Significant at 10% level

*5.4.3 Model specification test*

To check the reliability and ensure the robustness of findings, a model specification test was conducted to check whether the Tobit model was estimated using the right functional form and included the relevant explanatory variables. The test was conducted by using the *linktest* command in STATA 18 and the findings are reported in Table 8. The test included the predicted value of hat (*hat*) and its squared term (*hatsq*) as regressors. The findings shows that the squared term (*hatsq*) was statistically insignificant (p value is 0.915), indicating that the model was correctly specified, no incorrect functional form was used, and no relevant variables were omitted.

Table 8. Model specification test

| Variables | Coefficient | Standard Error | t | P > ǀtǀ |
| --- | --- | --- | --- | --- |
| hat | 0.902 | 0.431 | 2.09 | 0.037 |
| hatsq | 0.089 | 0.838 | 0.11 | 0.915 |
| constant | 0.026 | 0.015 | 1.73 | 0.085 |
| LR (chi2) | 49.589 | | | |
| Prob > chi2 | 0.000 | | | |

Source: Authors’ estimation, 2024.

*** Significant at 1% level, ** Significant at 5% level, *Significant at 10% level

“4. The manuscript states that all relevant data are provided in supporting files, but only the questionnaire is supplied, not the actual study dataset (see Data Availability section). This limits reproducibility and transparency.”

Response to comment 4:

“We have supplied data as supporting files in revised submission.”

“5. Treatment of missing data is insufficiently explained, as only the exclusion of two incomplete cases is mentioned (Lines 227–230). No further information is given on how other missing values or non-response were managed.”

Response to comment 5:

“Two households had partially missing responses, where 2–3 questions were not recorded by the data collectors for two sampled households. These missing values were minimal and handled by excluding the two households from analysis. This approach preserves the integrity of the dataset while maintaining a final sample size of 382 tea households. The manuscript has been updated to clarify this (**Lines 233–235)**

“A total of 384 tea households were initially surveyed. Due to partially missing information (2-3 question were not recorded by the data enumerators for two sampled households), responses from two households were completely excluded, resulting in a final sample size of 382 tea households, which closely aligns with the calculated sample size from Equation 1. Data collection took place between April and August, 2022.”

“6. English language issues are present throughout. For example, “this study`s” (Line 108) should be “this study's”; “Section 4 outlines discuss” (Line 133) should be “Section 4 discusses”; and “This result is available in consistent with” (Line 476) should be revised to “This result is consistent with.” The distinction between unidimensional and multidimensional poverty concepts is unclearly described (Lines 87–92), and terminology is not used consistently.””

Response to comment 6:

“We thank the reviewer for helping us to improve the writing. We corrected the mistakes in revised manuscript (track change file)

- study’s **(line 109)**
- we deleted the word ‘outline’ and converted ‘discuss’ into ‘discusses’ **(line 134)**
- We deleted the words ‘available in’. Now it is— ‘This result is consistent with’ **(line 495)**
- For maintaining consistence in terminologies, we add a word at **line number 88** and now the line is “Unidimensional energy poverty may indicate a household is energy poor in one aspect but does not necessarily reflect multidimensional energy poverty.””

At the end, Thanks a lot for your valuable feedback that helps to improve this papers’ quality. We expect that you will be satisfied from our response, but, still if you have any opinion, please, you are most warmly welcomed.

Thanks again!
