# Supplementary material for: Household level multidimensional energy poverty and its determinants: Evidence from tea estates in Bangladesh
Source: PLoS One. 2026 Aug 7;21(8):e0354309. doi: 10.1371/journal.pone.0354309 (PMC13450794; doi:10.1371/journal.pone.0354309)
Supplement: Supplementary file 5 [file pone.0354309.s006.docx]

**Response Letter to Editor**

Journal: PLOS ONE

Title: Household Level Multidimensional Energy Poverty and Its Determinants: Evidence from Tea Estates in Bangladesh

ID: PONE-D-25-43666R1

Dear Editor

Thank you so much for reviewing our manuscript and offer us with positive and detailed comments. We prepare following responses to explain what we have done in the revision. In order to help reviewers and editor to follow what we have revised and changed, we keep all changes tracked in the revised version, and mark responses in this response letter with color ‘blue’ and double quotation marks (“…”). All the black words are originally from review#2 that we cite with double citation mark and explanation of logic in response to comments but not changed in reviewed manuscript.

**Major comments:**

“1. Generalizability: The reliance on primary data from a single estate, while providing depth, may limit the generalizability of the findings to all tea estates in Bangladesh. A discussion of this limitation, along with the potential for future multi-site studies, would enhance the paper’s contextual framing. The study provides valuable insights into multidimensional energy poverty within tea estates in Bangladesh, with a focus on socioeconomic factors such as education, occupation, family size, and access to improved housing. While the research contributes to a nuanced understanding of energy deprivation in rural Bangladesh, the study could improve its global applicability. The use of primary data from a single estate limits generalizability to all tea estates in the country, and the paper would benefit from incorporating more detailed variables like appliance usage and electricity reliability. Some methodological choices, such as equal weights for MEPI dimensions and the 33.33% cutoff for energy poverty, are not sufficiently justified, which could raise questions about the robustness of the analysis. Further, addressing language and clarity issues in the manuscript could enhance its overall readability and impact. Despite these limitations, the paper makes a significant contribution to energy poverty literature, especially by focusing on tea workers, and offers practical policy recommendations that could improve energy access and socio-economic conditions in similar communities.”

Response to comment 1:

Thank you very much for your valuable and insightful comment. To deal with the generalizability issue we have added following lines in the revised manuscript as follows. Please find the addition between lines **121-130** (track changed revised manuscript) and lines **649-653** (track changed revised manuscript).

“Line 121-130: This research adopts a case-based analytical technique by concentrating on a single tea estate. Although reliance on one estate may restrict statistical generalizability to all tea estates in Bangladesh, it extensively examines household-level energy deprivation within a marginalized and policy-relevant community, and the findings remain still informative. Tea estates in Bangladesh administered under similar wage framework, institutional arrangements, housing facilities, patterns of energy access, and access to fundamental infrastructure. The findings therefore, provide context-specific insights into the mechanisms through which socioeconomic characteristics shape multidimensional energy poverty. These insights are meaningful for understanding energy deprivation in comparable tea labour communities in South Asia and other developing regions.”

“Line 649-653: this study has some limitations. Firstly, it is limited to a single tea estate, which may limit the generalization of findings. But the institutional and socioeconomic similarity of tea estates in Bangladesh indicates that the observed relationships are likely to hold in similar tea-labour based communities, making the findings analytically transferable rather than statistically representative.”

“2. Methodological Robustness: Some methodological choices—specifically, the use of equal weights for all dimensions of the Multidimensional Energy Poverty Index (MEPI) and the 33.33% cutoff threshold—require further justification. A more detailed rationale for these parameters would help address potential questions regarding the robustness of the analysis.”

Response to comment 2:

Thank you for helping in improving our paper by your insightful comment. We have added following lines in the revised manuscript as follows. Please find the addition between lines **313-323** (track changed revised manuscript) and lines **358-371** (track changed revised manuscript).

“Line 313-323: The application of equal weights represents both methodological clarity and the unavailability of a strong empirical or normative basis for allocating distinctive significance to particular dimensions. Cooking, lighting, and energy amenities indicate necessary and complementary aspects of household well-being, and emphasizing one dimension over another would introduce subjective value judgments. Therefore, equal weighting permits the poverty index to stay easily understandable, policy-neutral, and comparable to past MEPI based studies. Also, this approach has been extensively adopted in multidimensional poverty measurement when consensus on relative importance among dimensions is lacking. This approach assumes that each dimension contributes equally to overall deprivation and helps in minimizing subjective bias due to assigning unequal weights. Table 1 presents”

“Line 358-371: This study adopts the deprivation cut-off 33.33%, established by Alkire et al. [50], which has been widely used in past studies [47,59]. Given that each of the three dimensions carries equal weight, this cut-off implies that a household is categorized as energy poor if it is deprived in at least one dimension or an equivalent combination of indicators across dimensions. Conceptually, this cut-off minimizes the risk of under-identifying households facing considerable energy deprivation against the risk of overstating poverty due to marginal shortfalls. Moreover, previous studies have shown that MEPI estimates are generally robust to reasonable variations in the cutoff, typically within the 30–40% range [50,60]. Therefore, adopting the 33.33% cut-off ensures both conceptual consistency and comparability with the existing literature.”

“3. Variable Detail: The analysis could be enriched by incorporating more granular variables related to energy consumption, such as detailed appliance usage patterns and metrics for electricity reliability (e.g., frequency and duration of outages).”

Response to comment 3:

Thanks again for this crucial remark. To deal with the variable issue we have added following lines in the revised manuscript as follows. Please find the addition between lines **284-292** (track changed revised manuscript) and lines 653-669 (track changed revised manuscript).

“Line 284-292 The energy-related variables in this study are measured as access-based indicators rather than appliance-level usage or electricity service quality measures. Data on household ownership of electrical appliances, electricity consumption intensity, and the duration of power outages was not systematically collected during the household survey. This indicate the contextual characteristics of tea estate communities, where electricity use is limited to fundamental lighting and a small number of low-load appliances, with relatively little variation in appliance ownership across households. Consequently, the lighting dimension includes access to electricity rather than reliability or intensity of use.”

“Line 653-659: Secondly, the analysis depends on access-based indicators of energy amenities and does not incorporate appliance-level electricity usage or electricity reliability measures such as the frequency and duration of power outages. These aspects can affect household well-being and effective energy use but were beyond the scope of the present household survey. Consequently, the lighting dimension mainly represents electricity availability rather than service quality or utilization intensity. Future research could strengthen the evaluation of multidimensional energy poverty by incorporating information on appliance ownership, electricity usage patterns, and supply reliability to provide a more comprehensive measure of functional energy access. Despite these limitations, this study provides a robust baseline assessment of energy poverty in tea estate communities, where cooking-related energy poverty remains the dominant challenge.”

“4. Clarity and Language: A thorough review to address minor language and clarity issues would enhance the manuscript's overall readability and professional presentation for a global academic audience.

Response to comment 4:

To address language issues and grammatical errors we have made following changes in the manuscript. You will find them in several lines in the track changed revised manuscript.

“Line 29: (MEPI)”

“Line 242: was”

“Line 300: considering”

“Line 322: dimension”

**N.B.: During technical check we have asked to do the following**. “1. Your ethics statement should only appear in the Methods section of your manuscript. If your ethics statement is written in any section besides the Methods, please move it to the Methods section and delete it from any other section. Please ensure that your ethics statement is included in your manuscript, as the ethics statement entered into the online submission form will not be published alongside your manuscript.”

**Response:**

We would like to draw your kind attention that we did not write any text regrading ethical statement in any section of our manuscript except methods. To include the ethics statement in our manuscript we have deleted a line “The verbal consent of the respondents was taken at the beginning of the survey.” [you will find at line number **240-241** in our revised manuscript track change version]. Instead we have created a subsection and added the following text in the manuscript that you can track at line number **246-255** in our revised manuscript track change version.

*“Line 246-255:*

4.1.1. Ethical statement

Ethical approval for this research was obtained from the Sylhet Agricultural University Research System (SAURES), Sylhet Agricultural University, Bangladesh. The ethics committee endorsed the utilization of verbal informed consent from respondents of the study area due to the their lower level of educational background. Prior to each interview, respondents were enlightened about the study objectives, data collection procedures, voluntary participation, and confidentiality safeguards. Verbal consent was obtained from each respondents and documented by the data enumerator in the questionnaire. No identifying information was gathered, and all data were anonymized before analysis to ensure confidentiality.”

At the end, Thanks a lot for your valuable feedback that helps to improve this papers’ quality. We expect that you will be satisfied from our response, but, still if you have any opinion, please, you are most warmly welcomed.

Thanks again!
