# Supplementary material for: Household level multidimensional energy poverty and its determinants: Evidence from tea estates in Bangladesh
Source: PLoS One. 2026 Aug 7;21(8):e0354309. doi: 10.1371/journal.pone.0354309 (PMC13450794; doi:10.1371/journal.pone.0354309)
Supplement: Supplementary file 6 [file pone.0354309.s007.docx]

**Response letter to reviewer#4**

**Journal:** PLOS One

**Revised Title:** Household level multidimensional energy poverty and its determinants: Evidence from tea estates in Bangladesh

**Manuscript ID:**  **PONE-D-25-43666R2**

Dear reviewer #4,

Thank you so much for reviewing our manuscript and offer us with positive and detailed comments. We prepare following responses to explain what we have done in the revision 4. In order to help reviewers and editor to follow what we have revised and changed, we keep all changes tracked in the revised version, and mark responses in this response letter with color ‘blue’ and double quotation marks (“…”). All the black words are originally from review#3 that we cite with double citation mark and explanation of logic in response to comments but not changed in reviewed manuscript.

**Specific comments:**

1. The background section does not provide detailed information about tea workers’ unique kind of poverty and plights stemmed from their ghettoized situation and bonded labor-owner relationships that restrict their social mobility and extreme level of extraction of labor resources in exchange for a sarcastic level of unfair wages and deprivation of basic needs.

Response to comments 1:

“Thank you very much for your valuable comment. In response to your comment we have added following text in background section that you will find at line number (**Line 148-158**)in the revised manuscript track change version.

“Tea labours in Bangladesh are one of the most historically marginalized communities. Most are descendants of workers brought to tea estates during the colonial period and continue to reside within estate settlements that are geographically and socially isolated from surrounding communities. Their livelihoods are highly dependent on tea estate employment, resulting a long lasting labour-management relationship that restricts occupational mobility and other employment opportunities. In spite of several wage revisions over the time, tea labourers continue to receive relatively low wages compared to national living costs and often experience several forms of deprivation regarding to housing quality, sanitation, education, healthcare, and access to modern energy services. For example, Koiry et al. [16] have characterized tea-worker households as a backward community facing continuous multidimensional poverty and social exclusion.”

2. The background section includes some information that needs to be verified. For example, the information about land ownership provisions for tea workers should be supported by authentic sources if there are any such provisions. Ideally, tea workers are not supposed to own any land in a tea estate. However, sometimes, tea garden authorities lease out some fellow lands to the workers for cultivating crops. This does not mean any ideas of land ownership that the tea workers are allowed in tea gardens. Please refer to the sources of information if there is any.

Response to comments 2:

“Thank you very much for helping us to improve the quality of the paper through your observations. In response to your comment we have added following text in background section that you will find at line number (**Line 171-177**) in the revised manuscript track change version.

“Some tea estates temporarily allocate small plots of fallow land to workers for subsistence cultivation. However, such arrangements do not confer legal ownership rights, and tea workers generally do not possess ownership claims over tea-estate land. Those workers who do not get land from estate authority usually receives food support in the form of rice or wheat.”

3. This is also misleading that tea workers have engagement in commercial activities and even in agriculture, fish cultivation, poultry, cow and goat rearing etc. that should be counted as alternative livelihood options. The occupation-wise sample distribution of this study does not also support this idea. Therefore, please refer to authentic sources of this information.

Response to comments 3:

“Thank you very much for this crucial observations. In response to your comment we have added following text in background section that you will find at line number (**Line 182-186**)in the revised manuscript track change version.

“Although tea labour constitutes the primary occupation for nearly all surveyed households, some workers often involve in supplementary income-generating activities such as seasonal vegetable cultivation, poultry rearing, fish production, crop cultivation or casual wage labour outside normal tea-estate working hours. These activities usually supplement rather than replace tea-estate employment.”

4. The intro and background do not provide sufficient reflection on the sources of energy. Even, literature review does not reflect anything on tea workers’ access to energy and their multidimensional poverty.

Response to comments 4:

Thanks again. We also think that this is probably the strongest observation. In response to this comment we have added following lines that you will find at the line numbers (**Line 186-196**)in the revised manuscript track change version.

“In terms of energy use, tea households depend on conventional biomass based sources like firewood, crop residues, and dung cake for cooking and heating [15,23]. The findings in Table 2 indicate that only 2% tea households had access to LPG in the study area, implying limited adoption of modern cooking fuels. This scenario may represent affordability constraints, limited availability of LPG supply facilities, and long-established cooking practices within tea-estate communities. As a result, energy deprivation remains a crucial dimension of poverty among tea-worker households.”

5. The methodology does not detail it out if the samples were selected randomly from a list of workers. Or the selection process just followed the ideas of selecting households through a systematic random sampling process.

Response to comments 5:

In response to this comment we have added following lines that you will find at the line numbers in the revised manuscript track change version.

**Line 262-268 :** “Finally, a total of 384 tea households were selected through simple random sampling technique. A complete list of tea-worker households obtained from the tea-estate authority served as the sampling frame, ensuring that each household had an equal probability of selection. The advantages of simplicity, population representation, applicability to a broader group, and impartiality, among others, make simple random sampling suitable for this study.”

6. Why an open-ended questionnaire has been used is not clarified, when a multidimensional poverty framework clearly indicates a question structure and response options.

Response to comments 6:

Thank you very much for this detailed scrutiny. We had actually confused two words ‘open ended’ and ‘structures’, and we thought both are synonymous. However, in response to this comment we have replace the word “open-ended” with “structured” that you will find in the revised manuscript track change version.

**Line 274:** “Open-ended” 🡪 structured

7. Contract types, i.e., permanent or temporary workers, could have been an important dependent variable as the workers’ income mostly depends on how they are contracted for their work in tea gardens. Moreover, access to forest resources for cooking fuel and heating room during winter could have been another variable. Again, access to rationing services provided by tea garden owners or other social safety net supports provided by government could have been another variable.

Response to comments 7:

We appreciate the reviewer’s valuable suggestion. We agree that employment contract status (permanent versus temporary workers), access to forest resources, rationing services, and government social safety net programs may influence household energy poverty. However, these variables were not systematically collected during the household survey and therefore could not be incorporated into the empirical model. Nevertheless, tea estate workers operate under broadly similar institutional arrangements regarding wages, housing, and access to estate-provided facilities. We have acknowledged this limitation in the revised manuscript recommended that future studies incorporate these institutional and welfare-related variables to provide a more comprehensive understanding of energy poverty among tea workers. You will find the correction at line numbers between (**Line 725-729** )in the revised manuscript track change version.

“Future research may also include employment contract type (permanent versus temporary workers), access to forest resources, rationing services provided by tea estates, and participation in government social safety net programmes. These institutional and welfare-related factors may affect household energy choices and energy deprivation but were beyond the scope of the present study.”

8. The energy poverty dimensions should have included not only accessing electricity but also the level of having uninterrupted services given the reality of managing the supply through load-shedding that mostly affects the rural areas. In that situation, what are the alternative sources for electricity could have been included. Is it solar panel or is there any other alternative?

Response to comments 8:

We thank the reviewer for this valuable observation. We quite agree that electricity reliability, including the frequency and duration of load shedding, represents an important dimension of energy poverty, particularly in rural areas. However, the household survey was primarily designed to measure access to energy services and did not collect detailed information on electricity reliability or service interruptions. Consequently, the lighting dimension was measured based on access to electricity rather than the continuity or quality of electricity supply.

Regarding alternative electricity sources, information on solar energy access was collected during the survey. Only a very small number of households reported using solar energy, while the overwhelming majority relied on the conventional electricity network. Given the extremely limited prevalence of alternative electricity technologies in the study area, these sources were not incorporated as a separate dimension in the multidimensional energy poverty assessment.

To address the reviewer’s concern, we have clarified this issue in the methodology and limitations sections of the revised manuscript and have highlighted the need for future studies to incorporate electricity reliability measures and alternative energy technologies to provide a more comprehensive assessment of energy poverty.

In response to this comment we have added following lines that you will find at the line numbers in the revised manuscript track change version.

**Line 330-334 :** “Yet a small number of surveyed households reported using solar energy for lighting, the majority depended on the traditional electricity network. Information on electricity reliability, including the frequency and duration of load shedding, was not collected. So, the lighting dimension captures access to electricity rather than the continuity or quality of electricity supply.’

**Line 718-723 :** ‘Furthermore, while a small number of households reported access to solar energy, alternative electricity technologies were uncommon in the study area. Therefore, future research can strengthen the evaluation of multidimensional energy poverty by incorporating electricity reliability indicators, involving load shedding frequency, duration, role of alternative energy sources such as solar home systems, information on appliance ownership, electricity usage patterns, and supply reliability to provide a more comprehensive measure of functional energy access.’”

9. In occupation-wise analysis, does it make sense to compare with other types of occupations, when the tea workers are 98.69%? Rather, the categories like full-time workers, part-time workers, full-time workers plus other income sources, part-time workers and other income sources could have made more sense. Table 5 and 7 underline this point as the occupation like farming, service and business are found to be not statistically significant in determining energy poverty in tea gardens. However, other occupations have been found to be significant, though, it has not been defined what it means by other occupations.

Response to comments 9:

We appreciate the reviewer's suggestion regarding a classification based on full-time and part-time employment status. Such a categorization could indeed provide additional insights into variations in energy poverty among tea-worker households. However, the household survey did not collect information on employment intensity (e.g., full-time versus part-time tea work) or the combination of tea work with secondary income-generating activities. Consequently, it was not possible to construct these alternative occupational categories without introducing assumptions beyond the available data.

The occupational variable was therefore classified according to the household's primary livelihood source, which is a commonly used approach in household socioeconomic analyses. To improve clarity, we have now explicitly defined the "other occupations" category and provided additional discussion of its characteristics in the revised manuscript.

We have made following changes in our manuscript that you will find at following line numbers in the revised manuscript track change version.

**Line 310-313:** “other occupations include casual wage labour outside tea estates, rickshaw pulling, firewood trading, transport-related work, and similar informal income-generating activities (coded as 4).”

**Line 551-553 :** “(occupations mainly consisted of casual and informal labour activities characterized by unstable earnings and limited access to employment-related benefits.)”

10. Are there any tea workers who have tertiary education and still work in tea garden and remain in poverty?

Response to comments 10:

“In response to this comment we have added following line that you will find at the line numbers in the revised manuscript track change version.

**Line 441-445 :** “Yet, tertiary education was found relatively uncommon (2%) in the study area suggesting that a small number of individuals with higher education stayed within tea-estate probably due to of family ties, limited employment opportunities, or engagement in supervisory and administrative activities related with tea-estate operations.”

11. Why income does not have significant implication in determining energy poverty should be revisited, because improved housing, number of earning members, occupations are closely linked with income. This finding may give a wrong message to the policymakers and tea garden owners regarding the long, undisputed and unfair low wages in the tea gardens of Bangladesh.

Response to comments 11:

“In response to this comment we have added following line that you will find at the line numbers 607-615 in the revised manuscript track change version.

**Line 607-615:** “However, the statistical insignificance of income should not be interpreted as evidence that household earnings are unimportant for reducing energy poverty. Instead, the impact of income may be indirectly captured by correlated socioeconomic factors such as housing quality, land ownership, occupational status, and the number of earning members, all of which represents underlying household welfare. This indicates that income does not exert an additional independent effect once these correlated characteristics are controlled for in the model. Nevertheless, income-generating opportunities and wage improvements remain central to addressing energy deprivation among tea workers. These results also”

12. This study underlines the importance of improving socioeconomic conditions of tea workers to help decrease their energy poverty but does not reflect on the issues of other practical dimensions of tea workers’ poverty that are associated with their subjugation, bonded labor and ghetto situation.

Response to comments 12:

“In response to this comment we have added following line that you will find at the line numbers 685-694 in the revised manuscript track change version.

**Line 685-694 :** The perseverance of energy poverty among tea workers should be interpreted within the historical and socioeconomic structure of tea estates in Bangladesh. Tea workers have historically faced social marginalization, limited upward mobility, and restricted livelihood diversification, with sustained dependence on estate-based employment systems. These structural constraints reduce household ability to accept cleaner and more reliable energy sources, even where basic energy infrastructure is available. Consequently, addressing energy poverty in this context requires more improvements in physical access that strengthen social inclusion, labour welfare, income stability, and overall living conditions within tea-growing communities.

13. This paper requires a thorough revision to address these observations.

Response to comments 13:

We think that the responses and corrections based on all crucial and valuable comments from you satisfy this comment (13). Thank you !

*** We think these responses also fulfill your overall comments.

At the end, Thanks a lot for your valuable feedback that helps to improve this papers’ quality. We expect that you will be satisfied from our response, but, still if you have any opinion, please, you are most warmly welcomed.

Thanks again!
