# Supplementary material for: Household level multidimensional energy poverty and its determinants: Evidence from tea estates in Bangladesh
Source: PLoS One. 2026 Aug 7;21(8):e0354309. doi: 10.1371/journal.pone.0354309 (PMC13450794; doi:10.1371/journal.pone.0354309)
Supplement: Peer review history

# Peer review: Decision Letter 0

**DOI:** 10.1371/journal.pone.0354309.r001

**Authors:** Rajan Parajuli

11 Sep 2025

PONE-D-25-43666

Household Level Multidimensional Energy Poverty And Its Determinants: Evidence From Tea Estates In Bangladesh

PLOS ONE

Dear Dr. Farid,

Thank you for submitting your manuscript to PLOS ONE. After careful consideration, we feel that it has merit but does not fully meet PLOS ONE’s publication criteria as it currently stands. Therefore, we invite you to submit a revised version of the manuscript that addresses the points raised during the review process.

Please submit your revised manuscript by Oct 26 2025 11:59PM. If you will need more time than this to complete your revisions, please reply to this message or contact the journal office at plosone@plos.org. When you're ready to submit your revision, log on to https://www.editorialmanager.com/pone/ and select the 'Submissions Needing Revision' folder to locate your manuscript file.

Please include the following items when submitting your revised manuscript:

- A rebuttal letter that responds to each point raised by the academic editor and reviewer(s). You should upload this letter as a separate file labeled 'Response to Reviewers'.
- A marked-up copy of your manuscript that highlights changes made to the original version. You should upload this as a separate file labeled 'Revised Manuscript with Track Changes'.
- An unmarked version of your revised paper without tracked changes. You should upload this as a separate file labeled 'Manuscript'.

If you would like to make changes to your financial disclosure, please include your updated statement in your cover letter. Guidelines for resubmitting your figure files are available below the reviewer comments at the end of this letter.

If applicable, we recommend that you deposit your laboratory protocols in protocols.io to enhance the reproducibility of your results. Protocols.io assigns your protocol its own identifier (DOI) so that it can be cited independently in the future. For instructions see: https://journals.plos.org/plosone/s/submission-guidelines#loc-laboratory-protocols. Additionally, PLOS ONE offers an option for publishing peer-reviewed Lab Protocol articles, which describe protocols hosted on protocols.io. Read more information on sharing protocols at https://plos.org/protocols?utm_medium=editorial-email&utm_source=authorletters&utm_campaign=protocols.

We look forward to receiving your revised manuscript.

Kind regards,

Rajan Parajuli

Academic Editor

PLOS ONE

Journal Requirements:

When submitting your revision, we need you to address these additional requirements.

1. Please ensure that your manuscript meets PLOS ONE's style requirements, including those for file naming. The PLOS ONE style templates can be found at https://journals.plos.org/plosone/s/file?id=wjVg/PLOSOne_formatting_sample_main_body.pdf and https://journals.plos.org/plosone/s/file?id=ba62/PLOSOne_formatting_sample_title_authors_affiliations.pdf

2. In the ethics statement in the Methods, you have specified that verbal consent was obtained. Please provide additional details regarding how this consent was documented and witnessed, and state whether this was approved by the IRB.

3. When completing the data availability statement of the submission form, you indicated that you will make your data available on acceptance. We strongly recommend all authors decide on a data sharing plan before acceptance, as the process can be lengthy and hold up publication timelines. Please note that, though access restrictions are acceptable now, your entire data will need to be made freely accessible if your manuscript is accepted for publication. This policy applies to all data except where public deposition would breach compliance with the protocol approved by your research ethics board. If you are unable to adhere to our open data policy, please kindly revise your statement to explain your reasoning and we will seek the editor's input on an exemption. Please be assured that, once you have provided your new statement, the assessment of your exemption will not hold up the peer review process.

4. We note that the grant information you provided in the ‘Funding Information’ and ‘Financial Disclosure’ sections do not match.

When you resubmit, please ensure that you provide the correct grant numbers for the awards you received for your study in the ‘Funding Information’ section.

5. Please amend either the title on the online submission form (via Edit Submission) or the title in the manuscript so that they are identical.

6. We note that Figure 1 in your submission contain [map/satellite] images which may be copyrighted. All PLOS content is published under the Creative Commons Attribution License (CC BY 4.0), which means that the manuscript, images, and Supporting Information files will be freely available online, and any third party is permitted to access, download, copy, distribute, and use these materials in any way, even commercially, with proper attribution. For these reasons, we cannot publish previously copyrighted maps or satellite images created using proprietary data, such as Google software (Google Maps, Street View, and Earth). For more information, see our copyright guidelines: http://journals.plos.org/plosone/s/licenses-and-copyright.

We require you to either (1) present written permission from the copyright holder to publish these figures specifically under the CC BY 4.0 license, or (2) remove the figures from your submission:

a. You may seek permission from the original copyright holder of Figure 1 to publish the content specifically under the CC BY 4.0 license.

We recommend that you contact the original copyright holder with the Content Permission Form (http://journals.plos.org/plosone/s/file?id=7c09/content-permission-form.pdf) and the following text:

“I request permission for the open-access journal PLOS ONE to publish XXX under the Creative Commons Attribution License (CCAL) CC BY 4.0 (http://creativecommons.org/licenses/by/4.0/). Please be aware that this license allows unrestricted use and distribution, even commercially, by third parties. Please reply and provide explicit written permission to publish XXX under a CC BY license and complete the attached form.”

Please upload the completed Content Permission Form or other proof of granted permissions as an "Other" file with your submission.

In the figure caption of the copyrighted figure, please include the following text: “Reprinted from [ref] under a CC BY license, with permission from [name of publisher], original copyright [original copyright year].”

b. If you are unable to obtain permission from the original copyright holder to publish these figures under the CC BY 4.0 license or if the copyright holder’s requirements are incompatible with the CC BY 4.0 license, please either i) remove the figure or ii) supply a replacement figure that complies with the CC BY 4.0 license. Please check copyright information on all replacement figures and update the figure caption with source information. If applicable, please specify in the figure caption text when a figure is similar but not identical to the original image and is therefore for illustrative purposes only.

The following resources for replacing copyrighted map figures may be helpful:

USGS National Map Viewer (public domain): http://viewer.nationalmap.gov/viewer/

The Gateway to Astronaut Photography of Earth (public domain): http://eol.jsc.nasa.gov/sseop/clickmap/

Maps at the CIA (public domain): https://www.cia.gov/library/publications/the-world-factbook/index.html and https://www.cia.gov/library/publications/cia-maps-publications/index.html

NASA Earth Observatory (public domain): http://earthobservatory.nasa.gov/

Landsat: http://landsat.visibleearth.nasa.gov/

USGS EROS (Earth Resources Observatory and Science (EROS) Center) (public domain): http://eros.usgs.gov/#

Natural Earth (public domain): http://www.naturalearthdata.com/

7. Please include a caption for figure 1.

8. If the reviewer comments include a recommendation to cite specific previously published works, please review and evaluate these publications to determine whether they are relevant and should be cited. There is no requirement to cite these works unless the editor has indicated otherwise.

Additional Editor Comments :

AE: both reviewers raised interesting and useful concerns and provided suggestions in the current version. I encourage authors to address and incorporate the review comments carefully. I look forward to the revised version.

[Note: HTML markup is below. Please do not edit.]

Reviewers' comments:

Reviewer's Responses to Questions

**Comments to the Author**

1. Is the manuscript technically sound, and do the data support the conclusions?

The manuscript must describe a technically sound piece of scientific research with data that supports the conclusions. Experiments must have been conducted rigorously, with appropriate controls, replication, and sample sizes. The conclusions must be drawn appropriately based on the data presented.

Reviewer #1: Yes

Reviewer #2: Partly

**********

2. Has the statistical analysis been performed appropriately and rigorously?

Reviewer #1: Yes

Reviewer #2: No

**********

3. Have the authors made all data underlying the findings in their manuscript fully available?

The PLOS Data policy requires authors to make all data underlying the findings described in their manuscript fully available without restriction, with rare exception (please refer to the Data Availability Statement in the manuscript PDF file). The data should be provided as part of the manuscript or its supporting information, or deposited to a public repository. For example, in addition to summary statistics, the data points behind means, medians and variance measures should be available. If there are restrictions on publicly sharing data—e.g. participant privacy or use of data from a third party—those must be specified.

Reviewer #1: Yes

Reviewer #2: No

**********

4. Is the manuscript presented in an intelligible fashion and written in standard English?

PLOS ONE does not copyedit accepted manuscripts, so the language in submitted articles must be clear, correct, and unambiguous. Any typographical or grammatical errors should be corrected at revision, so please note any specific errors here.

Reviewer #1: Yes

Reviewer #2: No

**********

5. Review Comments to the Author

Please use the space provided to explain your answers to the questions above. You may also include additional comments for the author, including concerns about dual publication, research ethics, or publication ethics. (Please upload your review as an attachment if it exceeds 20,000 characters)

Reviewer #1: This paper deals with measuring energy poverty and its determinants in tea estates in Bangladesh. The study is based on primary data collected for the specific study. The paper may be informative in the context of Bangladesh. However, the quality of paper needs to be improved for larger global audience. Following are few specific comments about the paper:

Since the data was collected by the authors, I feel that some of the important aspects could have been collected carefully. For e.g. whether energy poverty is due to access or affordability. If there is no reliable supply of LPG, it is natural that deprivation is due to access and not due to affordability alone. The authors may know the ground situation of the study area and incorporate the same in the paper.

Authors could have collected information on other variables like using household appliances using electricity to strengthen the measurement of electricity access. Further, information on its reliability could have been collected. These variables could be added to the lighting index as well. If this information is available, authors may incorporate the same.

Check the age code in page 10 for those with 25 to 54 years.

Why don’t you mention the variables in Table 2 instead of the codes used in the model. For example, age categories are mentioned instead of actual age group. There is nothing wrong but the reader will have to remember the codes mentioned earlier or frequently go back and forth to check what the category. It will affect the ease of reading the paper. Same thing applied for other categories as well.

Table 2 can be made more informative. For example, instead of just presenting sample’s frequency distribution, why not cross tab it with different dimensions of energy poverty and with the overall energy poverty score. That will give a lot more information than the present one.

Authors could have presented a detailed account of deprivation as seen in Sadath and Acharya (2017). For example, giving a detailed picture of extent of deprivation of the households across deciles or some other measure.

What is the need to make sense of insignificant coefficients about the result?

In line number 496 on page 26 there is ‘15’ number mentioned. Is it citation or something else? If citation, it is necessary to put it in bracket.

Let the recommendations of the study stem from the findings and not well-intentioned statements. For e.g. work-life balance, equal wage, etc.

Overall, the study examines a socially sensitive issue and redressal of the same has micro, macro and global implications. However, the paper falls short in making a compelling case for itself. I feel there is lot of scope for improvement. Authors may take the findings sportively and improve the paper.

Reviewer #2: Generalizability concerns arise because the sample is drawn solely from the Chatlapore tea estate (see Lines 207–217), yet the title and abstract imply a national scope. This restricts the ability to generalize findings to all tea estates in Bangladesh.

Methodological justification is lacking for several key decisions. The use of equal weights for MEPI dimensions is presented without empirical or theoretical support (Lines 276–279). The 33.33% poverty cutoff is adopted from Alkire et al. without sensitivity analysis or rationale for its appropriateness (Lines 308–309).

Robustness and model checks are missing. No alternative specifications or tests for multicollinearity among categorical variables are presented (see description of model variables, Lines 424–431 and Table 5). There are also no diagnostic tests for Tobit assumptions reported in the results section.

The manuscript states that all relevant data are provided in supporting files, but only the questionnaire is supplied, not the actual study dataset (see Data Availability section). This limits reproducibility and transparency.

Treatment of missing data is insufficiently explained, as only the exclusion of two incomplete cases is mentioned (Lines 227–230). No further information is given on how other missing values or non-response were managed.

English language issues are present throughout. For example, “this study`s” (Line 108) should be “this study's”; “Section 4 outlines discuss” (Line 133) should be “Section 4 discusses”; and “This result is available in consistent with” (Line 476) should be revised to “This result is consistent with.” The distinction between unidimensional and multidimensional poverty concepts is unclearly described (Lines 87–92), and terminology is not used consistently.

To improve, the manuscript needs to either acknowledge its single-estate scope or broaden sampling; justify weighting and cutoff choices with sensitivity analysis; provide complete model diagnostics; share underlying data; clarify missing data handling; and ensure language and terminology are accurate and consistent throughout.

**********

6. PLOS authors have the option to publish the peer review history of their article (what does this mean?). If published, this will include your full peer review and any attached files.

If you choose “no”, your identity will remain anonymous but your review may still be made public.

**Do you want your identity to be public for this peer review?**  For information about this choice, including consent withdrawal, please see our Privacy Policy.

Reviewer #1: No

Reviewer #2: No

**********

[NOTE: If reviewer comments were submitted as an attachment file, they will be attached to this email and accessible via the submission site. Please log into your account, locate the manuscript record, and check for the action link "View Attachments". If this link does not appear, there are no attachment files.]

While revising your submission, please upload your figure files to the Preflight Analysis and Conversion Engine (PACE) digital diagnostic tool, https://pacev2.apexcovantage.com/. PACE helps ensure that figures meet PLOS requirements. To use PACE, you must first register as a user. Registration is free. Then, login and navigate to the UPLOAD tab, where you will find detailed instructions on how to use the tool. If you encounter any issues or have any questions when using PACE, please email PLOS at figures@plos.org. Please note that Supporting Information files do not need this step.

# Author comment: Author response to Decision Letter 1

**DOI:** 10.1371/journal.pone.0354309.r002

6 Nov 2025

Response letter to reviewer#1

Journal: PLOS ONE

Title: Household Level Multidimensional Energy Poverty and Its Determinants: Evidence from Tea Estates in Bangladesh

ID: PONE-D-25-43666

Dear reviewer #1,

Thank you so much for reviewing our manuscript and offer us with positive and detailed comments. We prepare following responses to explain what we have done in the revision. In order to help reviewers and editor to follow what we have revised and changed, we keep all changes tracked in the revised version, and mark responses in this response letter with color ‘blue’ and double quotation marks (“…”). All the black words are originally from review#1 that we cite with double citation mark and explanation of logic in response to comments but not changed in reviewed manuscript.

Major comments:

“Overall comment: This paper deals with measuring energy poverty and its determinants in tea estates in Bangladesh. The study is based on primary data collected for the specific study. The paper may be informative in the context of Bangladesh. However, the quality of paper needs to be improved for larger global audience.”

Response to comment 1:

Thank you very much for your positive feedback and suggestions to improve the paper.

“1. Since the data was collected by the authors, I feel that some of the important aspects could have been collected carefully. For e.g. whether energy poverty is due to access or affordability. If there is no reliable supply of LPG, it is natural that deprivation is due to access and not due to affordability alone. The authors may know the ground situation of the study area and incorporate the same in the paper.”

Response to comment 1:

We thank the reviewer for this observation. In the study area, LPG supply points are located far from most households, requiring respondents to spend around 600 Bangladesh Taka for transportation to purchase LPG. This indicates that the primary cause of energy deprivation is limited access rather than affordability alone. We have clarified this in the revised manuscript, highlighting that physical distance to LPG outlets constrains usage, while the associated transportation cost adds a secondary financial burden. Our changes are documented (see line number: - number 298-303) in the revised manuscript (track change mode).

“In many parts of the study area, electricity infrastructure from the government or the estate was absent. So, households had no scope to use electricity. Likewise, LPG supply points were located far from most households, requiring them to spend around 5.55 US dollar (i.e., 600 Bangladesh Taka @ 1 US dollar = 108 Bangladesh Taka) on transportation to purchase LPG. These circumstances indicate that the primary cause of energy deprivation is limited access rather than affordability.”

“2. Authors could have collected information on other variables like using household appliances using electricity to strengthen the measurement of electricity access. Further, information on its reliability could have been collected. These variables could be added to the lighting index as well. If this information is available, authors may incorporate the same.”

Response to comment 2:

We thank the reviewer for the valuable suggestion. Unfortunately, our survey did not collect detailed information on household appliances or electricity reliability. We mainly focused on the availability of basic facilities. We acknowledge that including such variables could enhance the measurement of electricity access and the robustness of the lighting index. We have clearly noted this as a limitation in the revised manuscript (lines: - 619-623). We have also discussed the potential implications of this limitation for interpreting our results and suggested it as an avenue for future research.

“However, this study could not incorporate detailed information on household appliances due to respondents’ reluctance. This limitation may result in an incomplete evaluation of electricity access, as the lighting index mainly focuses on availability rather than usage. Future research could incorporate appliances ownership and reliability indicators to deliver a comprehensive assessment of electricity access.”

“3. Check the age code in page 10 for those with 25 to 54 years.”

Response to comment 3:

We also noticed missing codes for some variables and we added that in the revised manuscript. There were code mistakes according to your comment. Thank you for scrutinize comment. We corrected as follows (See lines 244-245; page 11 and lines: - 266-267, page 12 for the adding description of codes).

“25 to 54 years (coded as 2), and above 55 years (coded as 3). If households have access to these amenities, they are coded as 1; otherwise, coded as 0 for no access.”

“4. Why don’t you mention the variables in Table 2 instead of the codes used in the model. For example, age categories are mentioned instead of actual age group. There is nothing wrong but the reader will have to remember the codes mentioned earlier or frequently go back and forth to check what the category. It will affect the ease of reading the paper. Same thing applied for other categories as well.”

Response to comment 4:

“Thank you for your comment. We have included the actual names instead of code in our revised manuscript-track change mode. (see table 2). For ease we are not presenting here the table.

5. Table 2 can be made more informative. For example, instead of just presenting sample’s frequency distribution, why not cross tab it with different dimensions of energy poverty and with the overall energy poverty score. That will give a lot more information than the present one.”

Response to comment 5:

“We appreciate your suggestion regarding Table 2. The primary purpose of Table 2 is to provide a descriptive overview of the sample characteristics, which is standard practice in empirical studies. Cross-tabulating at this stage would conflate descriptive presentation with analytical results, potentially creating redundancy and obscuring clarity.

The relationships between household characteristics and energy poverty dimensions are systematically examined in subsequent tables and regression analyses (TOBIT), which provide a more rigorous and interpretable assessment. We think that, presenting cross-tabulations in the main descriptive table might not add substantial interpretative value but could complicate the presentation for readers.

Therefore, we maintain that the current format of Table 2 , which we think is methodologically appropriate and sufficient for conveying sample composition.”

“We hope our response will satisfy you.”

6. Authors could have presented a detailed account of deprivation as seen in Sadath and Acharya (2017). For example, giving a detailed picture of extent of deprivation of the households across deciles or some other measure. What is the need to make sense of insignificant coefficients about the result?”

Response to comment 6:

“We thank you for this observation and insightful comment. However, the objective of the present study was not to provide a descriptive decomposition of deprivation, but rather to empirically identify the key determinants influencing household deprivation. The type of analysis suggested—such as the distribution of deprivation across income deciles, as in Sadath and Acharya (2017)—is valuable for exploratory assessment, but it does not directly contribute to the estimation and interpretation of the determinants of deprivation, which is the central focus of this paper.

Moreover, the manuscript already includes descriptive statistics that summarize the extent and pattern of deprivation among the sampled households. Adding another level of descriptive breakdown might divert attention from its econometric focus.

Regarding the discussion of insignificant coefficients, these were not interpreted as meaningful effects, but were mentioned briefly to demonstrate that certain variables did not exhibit a statistically significant influence once other covariates were controlled for. We think, presenting and acknowledging insignificant results is a standard econometric practice, as it provides a complete picture of the model outcomes and helps to identify which factors are or are not empirically relevant.

For these reasons, we consider the current analytical structure can be appropriate for addressing the stated objectives of the study.”

“7. In line number 496 on page 26 there is ‘15’ number mentioned. Is it citation or something else? If citation, it is necessary to put it in bracket.”

Response to comment 7:

That one is typo error. We have deleted that. You will find the deletion on line number 514 at page number 27 in our revised manuscript (track change mode). Thank you.

“8. Let the recommendations of the study stem from the findings and not well-intentioned statements. For e.g. work-life balance, equal wage, etc.”

Response to comment 8:

“Thank you very much for these important observations. We have rearranged some lines, deleted some lines and rewritten the recommendation part in our conclusion section. You will find that in the revised manuscript (Track change mode: lines:- 602-618). However, we are presenting the change and new part here in below:”

“The study identifies higher energy poverty among female-headed households, attributable to their dual responsibilities of tea leaf plucking and household cooking. To address energy poverty effectively, the government could initiate housing schemes or partnerships with tea estates, where dependence on traditional cooking fuels is high, providing initial subsidies for LPG or establishing rural gas pipeline networks could alleviate energy poverty. Education plays an important role, underscoring the need for programs that promote education and awareness of clean energy technologies among tea workers. Finally, households with more earning members and large land holdings experience lower energy poverty, highlighting the potential of programs that increase household income through livelihood diversification, skill development or increased land-based productivity. Thus, improving housing, education, and clean energy access, especially for female-headed and larger households can effectively reduce multidimensional energy poverty in tea estates.”

Note that, changing (correcting) recommendation, signaled us to change recommendation in Abstract and we did so, which you will find in the lines between 35-38 in the revised manuscript (Track change).

”9. Overall, the study examines a socially sensitive issue and redressal of the same has micro, macro and global implications. However, the paper falls short in making a compelling case for itself. I feel there is lot of scope for improvement. Authors may take the findings sportively and improve the paper.”

Response to comment 9:

Thanks a lot for your comment. We have tried to improve our paper according to your feedback. Now, we hope that this paper have dealt enough with its shortcomings.

At the end, Thanks a lot for your valuable feedback that helps to improve this papers’ quality. We expect that you will be satisfied from our response, but, still if you have any opinion, please, you are most warmly welcomed.

Response letter to reviewer#2

Journal: PLOS ONE

Title: Household Level Multidimensional Energy Poverty and Its Determinants: Evidence from Tea Estates in Bangladesh

ID: PONE-D-25-43666

Dear reviewer #1,

Thank you so much for reviewing our manuscript and offer us with positive and detailed comments. We prepare following responses to explain what we have done in the revision. In order to help reviewers and editor to follow what we have revised and changed, we keep all changes tracked in the revised version, and mark responses in this response letter with color ‘blue’ and double quotation marks (“…”). All the black words are originally from review#1 that we cite with double citation mark and explanation of logic in response to comments but not changed in reviewed manuscript.

Major comments:

“1. Generalizability concerns arise because the sample is drawn solely from the Chatlapore tea estate (see Lines 207–217), yet the title and abstract imply a national scope. This restricts the ability to generalize findings to all tea estates in Bangladesh.”

Response to comment 1:

Thank you very much for this crucial observation. The Chatlapore tea estate was carefully selected because its socioeconomic and infrastructural characteristics closely resemble those of other tea estates in Bangladesh. The estate represents a typical case in terms of worker livelihood patterns, energy access, and institutional arrangements common across the tea sector. Therefore, while the study does not aim to make statistical generalizations to all tea estates, it provides analytical generalization and policy-relevant insights applicable to similar tea estate communities in Bangladesh. Furthermore, the objective of the paper is to understand the multidimensional nature of energy poverty within the tea estate context, not to make national-level estimates. Hence, the study’s scope and conclusions remain consistent with its design and contribution. However, we made the following changes in the lines 209-213 in revised manuscript (Track mode).

“The Chatlapore tea estate in the Lungla valley of the Moulvibazar district, Bangladesh was chosen as the study area to assess the multidimensional energy poverty. The demographic, infrastructural and institutional characteristics of this study area closely resemble to other tea estates in Bangladesh. For this reason, this study was concentrated in one tea estate. Moreover, this area was chosen because both the conventional and modern energy amenities are found in this area.”

“2. Methodological justification is lacking for several key decisions. The use of equal weights for MEPI dimensions is presented without empirical or theoretical support (Lines 276–279). The 33.33% poverty cutoff is adopted from Alkire et al. without sensitivity analysis or rationale for its appropriateness (Lines 308–309).”

Response to comment 2:

Thank you very much for providing positive review and comment. We have addressed your comment by following way –

““Line 287-290 (in revised manuscript) : Equal weighting is consistent with the approach of Alkire and Foster [48] and has been used in past studies such as Nussbaumer et al. [47] and Day et al. [59]. This approach assumes that each dimensions contributes equally to overall deprivation and remove subjective bias due to unequal weights.”

“Line 325-330 (in revised manuscript): This study adopts the 33.33% cut-off rate established by Alkire et al. [50], which has been widely used in past studies [47,59] to identify households experiencing deprivation across various dimensions and to determine both the headcount ratio and poverty intensity. Previous research also suggests that findings are generally robust to reasonable variations in the cutoff (e.g., 30–40%) [50, 60]. The choice of this benchmark allows for comparability with existing studies and provides a standard reference for interpreting energy poverty in the study area.””

“3. Robustness and model checks are missing. No alternative specifications or tests for multicollinearity among categorical variables are presented (see description of model variables, Lines 424–431 and Table 5). There are also no diagnostic tests for Tobit assumptions reported in the results section.”

Response to comment 3:

“Again, we want express our appreciation for your valuable comments to improve this paper. We have done several diagnostic tests and report them in a separate sub-section (5.4) under section 5. The name of subsection is 5.4. diagnostic test result. We mentioned the changes here in blue color text. The changes lies between lines 535-580.

“5.4 Diagnostic test results

5.4.1 Multicollinearity, Heteroskedasticity and Normality test

To ensure the reliability of the estimated Tobit model, several diagnostic tests were conducted. Table 6 presents the findings of the multicollinearity, heteroskedasticity and normality tests using the Variance Inflation Factor (VIF), Breusch–Pagan (BP), and Shapiro–Wilk methods, respectively. An Ordinary Least Square (OLS) regression was estimated before performing the VI

Attachment Submitted filename: Responses to Reviewer.docx

# Peer review: Decision Letter 1

**DOI:** 10.1371/journal.pone.0354309.r003

**Authors:** Riccardo Ciacci

4 Feb 2026

PONE-D-25-43666R1

Household level multidimensional energy poverty and its determinants: Evidence from tea estates in Bangladesh

PLOS One

Dear Dr. Farid,

Thank you for submitting your manuscript to PLOS ONE. After careful consideration, we feel that it has merit but does not fully meet PLOS ONE’s publication criteria as it currently stands. Therefore, we invite you to submit a revised version of the manuscript that addresses the points raised during the review process.

================================

Thank you for the opportunity to review your insightful study on multidimensional energy poverty within the tea estates of Bangladesh. Your research provides a valuable and nuanced examination of this critical issue, with a commendable focus on key socioeconomic factors like education, occupation, family size, and housing quality.

Your work makes a significant contribution to the literature, particularly by highlighting the specific challenges faced by tea estate workers. The practical policy recommendations you offer have the potential to meaningfully improve energy access and socioeconomic conditions in this and similar communities.

To further strengthen the manuscript’s impact and rigor, I would like to offer several constructive suggestions for your consideration:

Generalizability: The reliance on primary data from a single estate, while providing depth, may limit the generalizability of the findings to all tea estates in Bangladesh. A discussion of this limitation, along with the potential for future multi-site studies, would enhance the paper’s contextual framing.

Methodological Robustness: Some methodological choices—specifically, the use of equal weights for all dimensions of the Multidimensional Energy Poverty Index (MEPI) and the 33.33% cutoff threshold—require further justification. A more detailed rationale for these parameters would help address potential questions regarding the robustness of the analysis.

Variable Detail: The analysis could be enriched by incorporating more granular variables related to energy consumption, such as detailed appliance usage patterns and metrics for electricity reliability (e.g., frequency and duration of outages).

Clarity and Language: A thorough review to address minor language and clarity issues would enhance the manuscript's overall readability and professional presentation for a global academic audience.

In summary, your paper delivers an important and focused contribution. Addressing the points above would serve to solidify its methodological foundations, broaden its applicability, and maximize its scholarly impact.

================================

Please submit your revised manuscript by Mar 21 2026 11:59PM. If you will need more time than this to complete your revisions, please reply to this message or contact the journal office at plosone@plos.org. When you're ready to submit your revision, log on to https://www.editorialmanager.com/pone/ and select the 'Submissions Needing Revision' folder to locate your manuscript file.

Please include the following items when submitting your revised manuscript:

- A letter that responds to each point raised by the academic editor and reviewer(s). You should upload this letter as a separate file labeled 'Response to Reviewers'.
- A marked-up copy of your manuscript that highlights changes made to the original version. You should upload this as a separate file labeled 'Revised Manuscript with Track Changes'.
- An unmarked version of your revised paper without tracked changes. You should upload this as a separate file labeled 'Manuscript'.

If you would like to make changes to your financial disclosure, please include your updated statement in your cover letter. Guidelines for resubmitting your figure files are available below the reviewer comments at the end of this letter.

If applicable, we recommend that you deposit your laboratory protocols in protocols.io to enhance the reproducibility of your results. Protocols.io assigns your protocol its own identifier (DOI) so that it can be cited independently in the future. For instructions see: https://journals.plos.org/plosone/s/submission-guidelines#loc-laboratory-protocols. Additionally, PLOS ONE offers an option for publishing peer-reviewed Lab Protocol articles, which describe protocols hosted on protocols.io. Read more information on sharing protocols at https://plos.org/protocols?utm_medium=editorial-email&utm_source=authorletters&utm_campaign=protocols.

We look forward to receiving your revised manuscript.

Kind regards,

Riccardo Ciacci

Guest Editor

PLOS One

Journal Requirements:

If the reviewer comments include a recommendation to cite specific previously published works, please review and evaluate these publications to determine whether they are relevant and should be cited. There is no requirement to cite these works unless the editor has indicated otherwise.

[Note: HTML markup is below. Please do not edit.]

Reviewers' comments:

Reviewer's Responses to Questions

**Comments to the Author**

1. If the authors have adequately addressed your comments raised in a previous round of review and you feel that this manuscript is now acceptable for publication, you may indicate that here to bypass the “Comments to the Author” section, enter your conflict of interest statement in the “Confidential to Editor” section, and submit your "Accept" recommendation.

Reviewer #2: All comments have been addressed

Reviewer #3: All comments have been addressed

**********

2. Is the manuscript technically sound, and do the data support the conclusions?

The manuscript must describe a technically sound piece of scientific research with data that supports the conclusions. Experiments must have been conducted rigorously, with appropriate controls, replication, and sample sizes. The conclusions must be drawn appropriately based on the data presented.

Reviewer #2: Yes

Reviewer #3: Yes

**********

3. Has the statistical analysis been performed appropriately and rigorously?

Reviewer #2: Yes

Reviewer #3: Yes

**********

4. Have the authors made all data underlying the findings in their manuscript fully available?

The PLOS Data policy requires authors to make all data underlying the findings described in their manuscript fully available without restriction, with rare exception (please refer to the Data Availability Statement in the manuscript PDF file). The data should be provided as part of the manuscript or its supporting information, or deposited to a public repository. For example, in addition to summary statistics, the data points behind means, medians and variance measures should be available. If there are restrictions on publicly sharing data—e.g. participant privacy or use of data from a third party—those must be specified.

Reviewer #2: Yes

Reviewer #3: Yes

**********

5. Is the manuscript presented in an intelligible fashion and written in standard English?

PLOS ONE does not copyedit accepted manuscripts, so the language in submitted articles must be clear, correct, and unambiguous. Any typographical or grammatical errors should be corrected at revision, so please note any specific errors here.

Reviewer #2: Yes

Reviewer #3: Yes

**********

6. Review Comments to the Author

Please use the space provided to explain your answers to the questions above. You may also include additional comments for the author, including concerns about dual publication, research ethics, or publication ethics. (Please upload your review as an attachment if it exceeds 20,000 characters)

Reviewer #2: The authors have made substantial and constructive revisions in response to the comments raised in the previous round of review. Overall, the manuscript has improved notably in terms of methodological clarity, transparency, and presentation.

Key concerns regarding the scope and generalizability of the study have been addressed by clearly framing the analysis as analytically rather than statistically generalizable and by explicitly situating the findings within the context of tea estate communities. Methodological decisions related to the construction of the Multidimensional Energy Poverty Index—specifically the use of equal weighting and the 33.33% poverty cutoff—are now justified with appropriate references and discussion, allowing for comparability with existing literature.

The statistical analysis has been strengthened through the addition of diagnostic and robustness checks. The authors now report tests for multicollinearity, heteroskedasticity, normality, and model specification, and they have addressed heteroskedasticity using robust standard errors and bootstrap estimation. The inclusion of an alternative OLS specification showing consistency with the Tobit results further supports the robustness of the findings.

Concerns regarding data transparency and reproducibility have been satisfactorily resolved. The authors now indicate that the underlying dataset is provided as supporting information, in line with PLOS ONE’s data availability requirements. The treatment of missing data has also been clarified, with incomplete observations appropriately excluded and documented.

The manuscript’s language and presentation have improved, with many previously noted grammatical and terminological issues corrected. While minor language issues may remain, they no longer hinder comprehension or interpretation. The revised discussion and policy recommendations are more closely grounded in the empirical results and avoid overly general or normative claims.

I find no concerns related to research ethics, dual publication, or conflicts of interest. Overall, the authors have responded adequately to the previous review comments, and the manuscript now meets the technical and reporting standards expected for publication in PLOS ONE.

Reviewer #3: The study provides valuable insights into multidimensional energy poverty within tea estates in Bangladesh, with a focus on socioeconomic factors such as education, occupation, family size, and access to improved housing. While the research contributes to a nuanced understanding of energy deprivation in rural Bangladesh, the study could improve its global applicability. The use of primary data from a single estate limits generalizability to all tea estates in the country, and the paper would benefit from incorporating more detailed variables like appliance usage and electricity reliability. Some methodological choices, such as equal weights for MEPI dimensions and the 33.33% cutoff for energy poverty, are not sufficiently justified, which could raise questions about the robustness of the analysis. Further, addressing language and clarity issues in the manuscript could enhance its overall readability and impact. Despite these limitations, the paper makes a significant contribution to energy poverty literature, especially by focusing on tea workers, and offers practical policy recommendations that could improve energy access and socio-economic conditions in similar communities.

**********

7. PLOS authors have the option to publish the peer review history of their article (what does this mean?). If published, this will include your full peer review and any attached files.

If you choose “no”, your identity will remain anonymous but your review may still be made public.

**Do you want your identity to be public for this peer review?**  For information about this choice, including consent withdrawal, please see our Privacy Policy.

Reviewer #2: No

Reviewer #3: No

**********

[NOTE: If reviewer comments were submitted as an attachment file, they will be attached to this email and accessible via the submission site. Please log into your account, locate the manuscript record, and check for the action link "View Attachments". If this link does not appear, there are no attachment files.]

To ensure your figures meet our technical requirements, please review our figure guidelines: https://journals.plos.org/plosone/s/figures

You may also use PLOS’s free figure tool, NAAS, to help you prepare publication quality figures: https://journals.plos.org/plosone/s/figures#loc-tools-for-figure-preparation.

NAAS will assess whether your figures meet our technical requirements by comparing each figure against our figure specifications.

# Author comment: Author response to Decision Letter 2

**DOI:** 10.1371/journal.pone.0354309.r004

23 Feb 2026

Response Letter to Reviewer #2

Journal: PLOS ONE

Title: Household Level Multidimensional Energy Poverty and Its Determinants: Evidence from Tea Estates in Bangladesh

ID: PONE-D-25-43666R1

Dear reviewer #2,

Thank you so much for reviewing our manuscript and offer us with positive and detailed comments. We prepare following responses to explain what we have done in the revision. In order to help reviewers and editor to follow what we have revised and changed, we keep all changes tracked in the revised version, and mark responses in this response letter with color ‘blue’ and double quotation marks (“…”). All the black words are originally from review#2 that we cite with double citation mark and explanation of logic in response to comments but not changed in reviewed manuscript.

Comments:

“The authors have made substantial and constructive revisions in response to the comments raised in the previous round of review. Overall, the manuscript has improved notably in terms of methodological clarity, transparency, and presentation.

Key concerns regarding the scope and generalizability of the study have been addressed by clearly framing the analysis as analytically rather than statistically generalizable and by explicitly situating the findings within the context of tea estate communities. Methodological decisions related to the construction of the Multidimensional Energy Poverty Index—specifically the use of equal weighting and the 33.33% poverty cutoff—are now justified with appropriate references and discussion, allowing for comparability with existing literature.

The statistical analysis has been strengthened through the addition of diagnostic and robustness checks. The authors now report tests for multicollinearity, heteroskedasticity, normality, and model specification, and they have addressed heteroskedasticity using robust standard errors and bootstrap estimation. The inclusion of an alternative OLS specification showing consistency with the Tobit results further supports the robustness of the findings.

Concerns regarding data transparency and reproducibility have been satisfactorily resolved. The authors now indicate that the underlying dataset is provided as supporting information, in line with PLOS ONE’s data availability requirements. The treatment of missing data has also been clarified, with incomplete observations appropriately excluded and documented.

The manuscript’s language and presentation have improved, with many previously noted grammatical and terminological issues corrected. While minor language issues may remain, they no longer hinder comprehension or interpretation. The revised discussion and policy recommendations are more closely grounded in the empirical results and avoid overly general or normative claims.

I find no concerns related to research ethics, dual publication, or conflicts of interest. Overall, the authors have responded adequately to the previous review comments, and the manuscript now meets the technical and reporting standards expected for publication in PLOS ONE.”

Response to comment:

Thanks a lot for your valuable feedback in the Review 1 and assist us to improve the quality of our paper and helps in the acceptance. Thanks again!

Response Letter to Reviewer #3

Journal: PLOS ONE

Title: Household Level Multidimensional Energy Poverty and Its Determinants: Evidence from Tea Estates in Bangladesh

ID: PONE-D-25-43666R1

Dear reviewer #3,

Thank you so much for reviewing our manuscript and offer us with positive and detailed comments. We prepare following responses to explain what we have done in the revision. In order to help reviewers and editor to follow what we have revised and changed, we keep all changes tracked in the revised version, and mark responses in this response letter with color ‘blue’ and double quotation marks (“…”). All the black words are originally from review#2 that we cite with double citation mark and explanation of logic in response to comments but not changed in reviewed manuscript.

Major comments:

“The study provides valuable insights into multidimensional energy poverty within tea estates in Bangladesh, with a focus on socioeconomic factors such as education, occupation, family size, and access to improved housing. While the research contributes to a nuanced understanding of energy deprivation in rural Bangladesh, the study could improve its global applicability. The use of primary data from a single estate limits generalizability to all tea estates in the country, and the paper would benefit from incorporating more detailed variables like appliance usage and electricity reliability. Some methodological choices, such as equal weights for MEPI dimensions and the 33.33% cutoff for energy poverty, are not sufficiently justified, which could raise questions about the robustness of the analysis. Further, addressing language and clarity issues in the manuscript could enhance its overall readability and impact. Despite these limitations, the paper makes a significant contribution to energy poverty literature, especially by focusing on tea workers, and offers practical policy recommendations that could improve energy access and socio-economic conditions in similar communities.”

Response to comment 1:

Thank you very much for your valuable and insightful comment. For easier understanding we have breakdown your comments and tried to address them as our understanding.

“1.1. The study provides valuable insights into multidimensional energy poverty within tea estates in Bangladesh, with a focus on socioeconomic factors such as education, occupation, family size, and access to improved housing. While the research contributes to a nuanced understanding of energy deprivation in rural Bangladesh, the study could improve its global applicability. The use of primary data from a single estate limits generalizability to all tea estates in the country.””

To deal with the generalizability issue we have added following lines in the revised manuscript as follows. Please find the addition between lines 121-130 (track changed revised manuscript) and lines 649-653 (track changed revised manuscript).

“Line 121-130: This research adopts a case-based analytical technique by concentrating on a single tea estate. Although reliance on one estate may restrict statistical generalizability to all tea estates in Bangladesh, it extensively examines household-level energy deprivation within a marginalized and policy-relevant community, and the findings remain still informative. Tea estates in Bangladesh administered under similar wage framework, institutional arrangements, housing facilities, patterns of energy access, and access to fundamental infrastructure. The findings therefore, provide context-specific insights into the mechanisms through which socioeconomic characteristics shape multidimensional energy poverty. These insights are meaningful for understanding energy deprivation in comparable tea labor communities in South Asia and other developing regions.”

“Line 649-653: this study has some limitations. Firstly, it is limited to a single tea estate, which may limit the generalization of findings. But the institutional and socioeconomic similarity of tea estates in Bangladesh indicates that the observed relationships are likely to hold in similar tea-labor based communities, making the findings analytically transferable rather than statistically representative.”

“1.2. the paper would benefit from incorporating more detailed variables like appliance usage and electricity reliability.”

To deal with the variable issue we have added following lines in the revised manuscript as follows. Please find the addition between lines 284-292 (track changed revised manuscript) and lines 653-669 (track changed revised manuscript).

“Line 284-292: The energy-related variables in this study are measured as access-based indicators rather than appliance-level usage or electricity service quality measures. Data on household ownership of electrical appliances, electricity consumption intensity, and the duration of power outages was not systematically collected during the household survey. This indicate the contextual characteristics of tea estate communities, where electricity use is limited to fundamental lighting and a small number of low-load appliances, with relatively little variation in appliance ownership across households. Consequently, the lighting dimension includes access to electricity rather than reliability or intensity of use.”

“Line 653-669: Secondly, the analysis depends on access-based indicators of energy amenities and does not incorporate appliance-level electricity usage or electricity reliability measures such as the frequency and duration of power outages. These aspects can affect household well-being and effective energy use but were beyond the scope of the present household survey. Consequently, the lighting dimension mainly represents electricity availability rather than service quality or utilization intensity. Future research could strengthen the evaluation of multidimensional energy poverty by incorporating information on appliance ownership, electricity usage patterns, and supply reliability to provide a more comprehensive measure of functional energy access. Despite these limitations, this study provides a robust baseline assessment of energy poverty in tea estate communities, where cooking-related energy poverty remains the dominant challenge.”

“1.3. Some methodological choices, such as equal weights for MEPI dimensions and the 33.33% cutoff for energy poverty, are not sufficiently justified, which could raise questions about the robustness of the analysis.”

To deal with the methodological choice issue we have added following lines in the revised manuscript as follows. Please find the addition between lines 313-323 (track changed revised manuscript) and lines 358-371 (track changed revised manuscript).

“Line 313-323: The application of equal weights represents both methodological clarity and the unavailability of a strong empirical or normative basis for allocating distinctive significance to particular dimensions. Cooking, lighting, and energy amenities indicate necessary and complementary aspects of household well-being, and emphasizing one dimension over another would introduce subjective value judgments. Therefore, equal weighting permits the poverty index to stay easily understandable, policy-neutral, and comparable to past MEPI based studies. Also, this approach has been extensively adopted in multidimensional poverty measurement when consensus on relative importance among dimensions is lacking. This approach assumes that each dimension contributes equally to overall deprivation and helps in minimizing subjective bias due to assigning unequal weights. Table 1 presents”

“Line 358-371: This study adopts the deprivation cut-off 33.33%, established by Alkire et al. [50], which has been widely used in past studies [47,59]. Given that each of the three dimensions carries equal weight, this cut-off implies that a household is categorized as energy poor if it is deprived in at least one dimension or an equivalent combination of indicators across dimensions. Conceptually, this cut-off minimizes the risk of under-identifying households facing considerable energy deprivation against the risk of overstating poverty due to marginal shortfalls. Moreover, previous studies have shown that MEPI estimates are generally robust to reasonable variations in the cutoff, typically within the 30–40% range [50,60]. Therefore, adopting the 33.33% cut-off ensures both conceptual consistency and comparability with the existing literature.”

“1.4. Further, addressing language and clarity issues in the manuscript could enhance its overall readability and impact.”

To address language issues and grammatical errors we have made following changes in the manuscript. You will find them in several lines in the track changed revised manuscript.

“Line 29: (MEPI)”

“Line 242: was”

“Line 300: considering”

“Line 322: dimension”

“1.5. Despite these limitations, the paper makes a significant contribution to energy poverty literature, especially by focusing on tea workers, and offers practical policy recommendations that could improve energy access and socio-economic conditions in similar communities.”

Thank you very much for considering the importance of our work despite some major issues.

At the end, Thanks a lot for your valuable feedback that helps to improve this papers’ quality. We expect that you will be satisfied from our response, but, still if you have any opinion, please, you are most warmly welcomed.

Thanks again!

Attachment Submitted filename: Response letter to the Editor 19 February 2026.docx

# Peer review: Decision Letter 2

**DOI:** 10.1371/journal.pone.0354309.r005

**Authors:** Nitish Gogoi

10 Jun 2026

PONE-D-25-43666R2

Household level multidimensional energy poverty and its determinants: Evidence from tea estates in Bangladesh

PLOS One

Dear Dr. Farid,

Thank you for submitting your manuscript to PLOS ONE. After careful consideration, we feel that it has merit but does not fully meet PLOS ONE’s publication criteria as it currently stands. Therefore, we invite you to submit a revised version of the manuscript that addresses the points raised during the review process.

**ACADEMIC EDITOR'S COMMENTS:**

Although one of our reviewers has recommended accepting your article, based on other reviewers’ comments on your article, I suggest you carefully read and revise the points made by them. They have raised some crucial questions that need to be revisited and revised before we consider your paper for publications in our journal.

Please submit your revised manuscript by Jul 25 2026 11:59PM. If you will need more time than this to complete your revisions, please reply to this message or contact the journal office at plosone@plos.org. When you're ready to submit your revision, log on to https://www.editorialmanager.com/pone/ and select the 'Submissions Needing Revision' folder to locate your manuscript file.

Please include the following items when submitting your revised manuscript:

- A letter that responds to each point raised by the academic editor and reviewer(s). You should upload this letter as a separate file labeled 'Response to Reviewers'.
- A marked-up copy of your manuscript that highlights changes made to the original version. You should upload this as a separate file labeled 'Revised Manuscript with Track Changes'.
- An unmarked version of your revised paper without tracked changes. You should upload this as a separate file labeled 'Manuscript'.

If you would like to make changes to your financial disclosure, please include your updated statement in your cover letter. Guidelines for resubmitting your figure files are available below the reviewer comments at the end of this letter.

If applicable, we recommend that you deposit your laboratory protocols in protocols.io to enhance the reproducibility of your results. Protocols.io assigns your protocol its own identifier (DOI) so that it can be cited independently in the future. For instructions see: https://journals.plos.org/plosone/s/submission-guidelines#loc-laboratory-protocols. Additionally, PLOS ONE offers an option for publishing peer-reviewed Lab Protocol articles, which describe protocols hosted on protocols.io. Read more information on sharing protocols at https://plos.org/protocols?utm_medium=editorial-email&utm_source=authorletters&utm_campaign=protocols.

As the corresponding author, your ORCID iD is verified in the submission system and will appear in the published article. PLOS supports the use of ORCID, and we encourage all coauthors to register for an ORCID iD and use it as well. Please encourage your coauthors to verify their ORCID iD within the submission system before final acceptance, as unverified ORCID iDs will not appear in the published article. *Only*  the individual author can complete the verification step; PLOS staff *cannot*  verify ORCID iDs on behalf of authors.

We look forward to receiving your revised manuscript.

Kind regards,

Nitish Gogoi, Ph.D.

Academic Editor

PLOS One

Journal Requirements:

If the reviewer comments include a recommendation to cite specific previously published works, please review and evaluate these publications to determine whether they are relevant and should be cited. There is no requirement to cite these works unless the editor has indicated otherwise.

[Note: HTML markup is below. Please do not edit.]

Reviewers' comments:

Reviewer's Responses to Questions

**Comments to the Author**

1. If the authors have adequately addressed your comments raised in a previous round of review and you feel that this manuscript is now acceptable for publication, you may indicate that here to bypass the “Comments to the Author” section, enter your conflict of interest statement in the “Confidential to Editor” section, and submit your "Accept" recommendation.

Reviewer #2: All comments have been addressed

Reviewer #4: (No Response)

**********

2. Is the manuscript technically sound, and do the data support the conclusions?

The manuscript must describe a technically sound piece of scientific research with data that supports the conclusions. Experiments must have been conducted rigorously, with appropriate controls, replication, and sample sizes. The conclusions must be drawn appropriately based on the data presented.

Reviewer #2: Yes

Reviewer #4: Partly

**********

3. Has the statistical analysis been performed appropriately and rigorously?

Reviewer #2: Yes

Reviewer #4: Yes

**********

4. Have the authors made all data underlying the findings in their manuscript fully available?

The PLOS Data policy requires authors to make all data underlying the findings described in their manuscript fully available without restriction, with rare exception (please refer to the Data Availability Statement in the manuscript PDF file). The data should be provided as part of the manuscript or its supporting information, or deposited to a public repository. For example, in addition to summary statistics, the data points behind means, medians and variance measures should be available. If there are restrictions on publicly sharing data—e.g. participant privacy or use of data from a third party—those must be specified.

Reviewer #2: Yes

Reviewer #4: Yes

**********

5. Is the manuscript presented in an intelligible fashion and written in standard English?

PLOS ONE does not copyedit accepted manuscripts, so the language in submitted articles must be clear, correct, and unambiguous. Any typographical or grammatical errors should be corrected at revision, so please note any specific errors here.

Reviewer #2: Yes

Reviewer #4: Yes

**********

6. Review Comments to the Author

Please use the space provided to explain your answers to the questions above. You may also include additional comments for the author, including concerns about dual publication, research ethics, or publication ethics. (Please upload your review as an attachment if it exceeds 20,000 characters)

Reviewer #2: Thank you for the careful and constructive revision. The manuscript has improved substantially, and the concerns from the previous round(s) have now been adequately addressed in my opinion. The revised version now provides an appropriate discussion of the limits of generalizability of a single-estate study, clarifies the scope of the energy indicators, and offers a reasonable justification for the equal weighting scheme and the 33.33% cutoff used for the MEPI. The methods, ethics statement, and presentation are also clearer, and the conclusions are now better aligned with the evidence presented. A few minor language issues remain, but they do not affect interpretation. I therefore consider the manuscript acceptable for publication in its current form.

Reviewer #4: Overall Comments:

The authors portray a useful research work centering on the energy poverty of the tea workers and their families living in a ghettoized situation with restricted opportunities for social mobility and bare minimum basic services provided by tea estates or tea garden owners who treat the tea workers as their bonded laborers. A study on the tea estates in Bangladesh that captures the plights of tea workers and their family members is significant from different viewpoints. Therefore, the authors’ attempt to reflect on their struggle to manage energy poverty provides a timely contribution to the knowledge world. However, like their unconventional poverty due to their ghetto-like conditions and bonded labor-like status, their energy poverty deserves an unconventional study covering contextual dimensions of poverty. Their poverty stems from extreme level of manipulation of labor ownership and deprivation of minimum living wages and basic needs. Therefore, income poverty, resource poverty and energy poverty among the tea workers should have been navigated through a more flexible framework that allows the researchers to capture more nuanced and intersectional dimensions of poverty, which may not necessarily resemble to the experiences of different other marginalized communities in Bangladesh.

Specific comments:

1. The background section does not provide detailed information about tea workers’ unique kind of poverty and plights stemmed from their ghettoized situation and bonded labor-owner relationships that restrict their social mobility and extreme level of extraction of labor resources in exchange for a sarcastic level of unfair wages and deprivation of basic needs.

2. The background section includes some information that needs to be verified. For example, the information about land ownership provisions for tea workers should be supported by authentic sources if there are any such provisions. Ideally, tea workers are not supposed to own any land in a tea estate. However, sometimes, tea garden authorities lease out some fellow lands to the workers for cultivating crops. This does not mean any ideas of land ownership that the tea workers are allowed in tea gardens. Please refer to the sources of information if there is any.

3. This is also misleading that tea workers have engagement in commercial activities and even in agriculture, fish cultivation, poultry, cow and goat rearing etc. that should be counted as alternative livelihood options. The occupation-wise sample distribution of this study does not also support this idea. Therefore, please refer to authentic sources of this information.

4. The intro and background do not provide sufficient reflection on the sources of energy. Even, literature review does not reflect anything on tea workers’ access to energy and their multidimensional poverty.

5. The methodology does not detail it out if the samples were selected randomly from a list of workers. Or the selection process just followed the ideas of selecting households through a systematic random sampling process.

6. Why an open-ended questionnaire has been used is not clarified, when a multidimensional poverty framework clearly indicates a question structure and response options.

7. Contract types, i.e., permanent or temporary workers, could have been an important dependent variable as the workers’ income mostly depends on how they are contracted for their work in tea gardens. Moreover, access to forest resources for cooking fuel and heating room during winter could have been another variable. Again, access to rationing services provided by tea garden owners or other social safety net supports provided by government could have been another variable.

8. The energy poverty dimensions should have included not only accessing electricity but also the level of having uninterrupted services given the reality of managing the supply through loadshedding that mostly affects the rural areas. In that situation, what are the alternative sources for electricity could have been included. Is it solar panel or is there any other alternative?

9. In occupation-wise analysis, does it make sense to compare with other types of occupations, when the tea workers are 98.69%? Rather, the categories like full-time workers, part-time workers, full-time workers plus other income sources, part-time workers and other income sources could have made more sense. Table 5 and 7 underline this point as the occupation like farming, service and business are found to be not statistically significant in determining energy poverty in tea gardens. However, other occupations have been found to be significant, though, it has not been defined what it means by other occupations.

10. Are there any tea workers who have tertiary education and still work in tea garden and remain in poverty?

11. Why income does not have significant implication in determining energy poverty should be revisited, because improved housing, number of earning members, occupations are closely linked with income. This finding may give a wrong message to the policymakers and tea garden owners regarding the long, undisputed and unfair low wages in the tea gardens of Bangladesh.

12. This study underlines the importance of improving socioeconomic conditions of tea workers to help decrease their energy poverty but does not reflect on the issues of other practical dimensions of tea workers’ poverty that are associated with their subjugation, bonded labor and ghetto situation.

13. This paper requires a thorough revision to address these observations.

**********

7. PLOS authors have the option to publish the peer review history of their article (what does this mean?). If published, this will include your full peer review and any attached files.

If you choose “no”, your identity will remain anonymous but your review may still be made public.

**Do you want your identity to be public for this peer review?**  For information about this choice, including consent withdrawal, please see our Privacy Policy.

Reviewer #2: No

Reviewer #4: **Yes:** Abu Said Md Juel Miah

**********

[NOTE: If reviewer comments were submitted as an attachment file, they will be attached to this email and accessible via the submission site. Please log into your account, locate the manuscript record, and check for the action link "View Attachments". If this link does not appear, there are no attachment files.]

To ensure your figures meet our technical requirements, please review our figure guidelines: https://journals.plos.org/plosone/s/figures

You may also use PLOS’s free figure tool, NAAS, to help you prepare publication quality figures: https://journals.plos.org/plosone/s/figures#loc-tools-for-figure-preparation.

NAAS will assess whether your figures meet our technical requirements by comparing each figure against our figure specifications.

Attachment Submitted filename: PLONE feedback.docx

# Author comment: Author response to Decision Letter 3

**DOI:** 10.1371/journal.pone.0354309.r006

2 Jul 2026

Response letter to reviewer#4

Journal: PLOS One

Revised Title: Household level multidimensional energy poverty and its determinants: Evidence from tea estates in Bangladesh

Manuscript ID: PONE-D-25-43666R2

Dear reviewer #4,

Thank you so much for reviewing our manuscript and offer us with positive and detailed comments. We prepare following responses to explain what we have done in the revision 4. In order to help reviewers and editor to follow what we have revised and changed, we keep all changes tracked in the revised version, and mark responses in this response letter with color ‘blue’ and double quotation marks (“…”). All the black words are originally from review#3 that we cite with double citation mark and explanation of logic in response to comments but not changed in reviewed manuscript.

Specific comments:

1. The background section does not provide detailed information about tea workers’ unique kind of poverty and plights stemmed from their ghettoized situation and bonded labor-owner relationships that restrict their social mobility and extreme level of extraction of labor resources in exchange for a sarcastic level of unfair wages and deprivation of basic needs.

Response to comments 1:

“Thank you very much for your valuable comment. In response to your comment we have added following text in background section that you will find at line number (Line 148-158)in the revised manuscript track change version.

“Tea labours in Bangladesh are one of the most historically marginalized communities. Most are descendants of workers brought to tea estates during the colonial period and continue to reside within estate settlements that are geographically and socially isolated from surrounding communities. Their livelihoods are highly dependent on tea estate employment, resulting a long lasting labour-management relationship that restricts occupational mobility and other employment opportunities. In spite of several wage revisions over the time, tea labourers continue to receive relatively low wages compared to national living costs and often experience several forms of deprivation regarding to housing quality, sanitation, education, healthcare, and access to modern energy services. For example, Koiry et al. [16] have characterized tea-worker households as a backward community facing continuous multidimensional poverty and social exclusion.”

2. The background section includes some information that needs to be verified. For example, the information about land ownership provisions for tea workers should be supported by authentic sources if there are any such provisions. Ideally, tea workers are not supposed to own any land in a tea estate. However, sometimes, tea garden authorities lease out some fellow lands to the workers for cultivating crops. This does not mean any ideas of land ownership that the tea workers are allowed in tea gardens. Please refer to the sources of information if there is any.

Response to comments 2:

“Thank you very much for helping us to improve the quality of the paper through your observations. In response to your comment we have added following text in background section that you will find at line number (Line 171-177) in the revised manuscript track change version.

“Some tea estates temporarily allocate small plots of fallow land to workers for subsistence cultivation. However, such arrangements do not confer legal ownership rights, and tea workers generally do not possess ownership claims over tea-estate land. Those workers who do not get land from estate authority usually receives food support in the form of rice or wheat.”

3. This is also misleading that tea workers have engagement in commercial activities and even in agriculture, fish cultivation, poultry, cow and goat rearing etc. that should be counted as alternative livelihood options. The occupation-wise sample distribution of this study does not also support this idea. Therefore, please refer to authentic sources of this information.

Response to comments 3:

“Thank you very much for this crucial observations. In response to your comment we have added following text in background section that you will find at line number (Line 182-186)in the revised manuscript track change version.

“Although tea labour constitutes the primary occupation for nearly all surveyed households, some workers often involve in supplementary income-generating activities such as seasonal vegetable cultivation, poultry rearing, fish production, crop cultivation or casual wage labour outside normal tea-estate working hours. These activities usually supplement rather than replace tea-estate employment.”

4. The intro and background do not provide sufficient reflection on the sources of energy. Even, literature review does not reflect anything on tea workers’ access to energy and their multidimensional poverty.

Response to comments 4:

Thanks again. We also think that this is probably the strongest observation. In response to this comment we have added following lines that you will find at the line numbers (Line 186-196)in the revised manuscript track change version.

“In terms of energy use, tea households depend on conventional biomass based sources like firewood, crop residues, and dung cake for cooking and heating [15,23]. The findings in Table 2 indicate that only 2% tea households had access to LPG in the study area, implying limited adoption of modern cooking fuels. This scenario may represent affordability constraints, limited availability of LPG supply facilities, and long-established cooking practices within tea-estate communities. As a result, energy deprivation remains a crucial dimension of poverty among tea-worker households.”

5. The methodology does not detail it out if the samples were selected randomly from a list of workers. Or the selection process just followed the ideas of selecting households through a systematic random sampling process.

Response to comments 5:

In response to this comment we have added following lines that you will find at the line numbers in the revised manuscript track change version.

Line 262-268 : “Finally, a total of 384 tea households were selected through simple random sampling technique. A complete list of tea-worker households obtained from the tea-estate authority served as the sampling frame, ensuring that each household had an equal probability of selection. The advantages of simplicity, population representation, applicability to a broader group, and impartiality, among others, make simple random sampling suitable for this study.”

6. Why an open-ended questionnaire has been used is not clarified, when a multidimensional poverty framework clearly indicates a question structure and response options.

Response to comments 6:

Thank you very much for this detailed scrutiny. We had actually confused two words ‘open ended’ and ‘structures’, and we thought both are synonymous. However, in response to this comment we have replace the word “open-ended” with “structured” that you will find in the revised manuscript track change version.

Line 274: “Open-ended” structured

7. Contract types, i.e., permanent or temporary workers, could have been an important dependent variable as the workers’ income mostly depends on how they are contracted for their work in tea gardens. Moreover, access to forest resources for cooking fuel and heating room during winter could have been another variable. Again, access to rationing services provided by tea garden owners or other social safety net supports provided by government could have been another variable.

Response to comments 7:

We appreciate the reviewer’s valuable suggestion. We agree that employment contract status (permanent versus temporary workers), access to forest resources, rationing services, and government social safety net programs may influence household energy poverty. However, these variables were not systematically collected during the household survey and therefore could not be incorporated into the empirical model. Nevertheless, tea estate workers operate under broadly similar institutional arrangements regarding wages, housing, and access to estate-provided facilities. We have acknowledged this limitation in the revised manuscript recommended that future studies incorporate these institutional and welfare-related variables to provide a more comprehensive understanding of energy poverty among tea workers. You will find the correction at line numbers between (Line 725-729 )in the revised manuscript track change version.

“Future research may also include employment contract type (permanent versus temporary workers), access to forest resources, rationing services provided by tea estates, and participation in government social safety net programmes. These institutional and welfare-related factors may affect household energy choices and energy deprivation but were beyond the scope of the present study.”

8. The energy poverty dimensions should have included not only accessing electricity but also the level of having uninterrupted services given the reality of managing the supply through load-shedding that mostly affects the rural areas. In that situation, what are the alternative sources for electricity could have been included. Is it solar panel or is there any other alternative?

Response to comments 8:

We thank the reviewer for this valuable observation. We quite agree that electricity reliability, including the frequency and duration of load shedding, represents an important dimension of energy poverty, particularly in rural areas. However, the household survey was primarily designed to measure access to energy services and did not collect detailed information on electricity reliability or service interruptions. Consequently, the lighting dimension was measured based on access to electricity rather than the continuity or quality of electricity supply.

Regarding alternative electricity sources, information on solar energy access was collected during the survey. Only a very small number of households reported using solar energy, while the overwhelming majority relied on the conventional electricity network. Given the extremely limited prevalence of alternative electricity technologies in the study area, these sources were not incorporated as a separate dimension in the multidimensional energy poverty assessment.

To address the reviewer’s concern, we have clarified this issue in the methodology and limitations sections of the revised manuscript and have highlighted the need for future studies to incorporate electricity reliability measures and alternative energy technologies to provide a more comprehensive assessment of energy poverty.

In response to this comment we have added following lines that you will find at the line numbers in the revised manuscript track change version.

Line 330-334 : “Yet a small number of surveyed households reported using solar energy for lighting, the majority depended on the traditional electricity network. Information on electricity reliability, including the frequency and duration of load shedding, was not collected. So, the lighting dimension captures access to electricity rather than the continuity or quality of electricity supply.’

Line 718-723 : ‘Furthermore, while a small number of households reported access to solar energy, alternative electricity technologies were uncommon in the study area. Therefore, future research can strengthen the evaluation of multidimensional energy poverty by incorporating electricity reliability indicators, involving load shedding frequency, duration, role of alternative energy sources such as solar home systems, information on appliance ownership, electricity usage patterns, and supply reliability to provide a more comprehensive measure of functional energy access.’”

9. In occupation-wise analysis, does it make sense to compare with other types of occupations, when the tea workers are 98.69%? Rather, the categories like full-time workers, part-time workers, full-time workers plus other income sources, part-time workers and other income sources could have made more sense. Table 5 and 7 underline this point as the occupation like farming, service and business are found to be not statistically significant in determining energy poverty in tea gardens. However, other occupations have been found to be significant, though, it has not been defined what it means by other occupations.

Response to comments 9:

We appreciate the reviewer's suggestion regarding a classification based on full-time and part-time employment status. Such a categorization could indeed provide additional insights into variations in energy poverty among tea-worker households. However, the household survey did not collect information on employment intensity (e.g., full-time versus part-time tea work) or the combination of tea work with secondary income-generating activities. Consequently, it was not possible to construct these alternative occupational categories without introducing assumptions beyond the available data.

The occupational variable was therefore classified according to the household's primary livelihood source, which is a commonly used approach in household socioeconomic analyses. To improve clarity, we have now explicitly defined the "other occupations" category and provided additional discussion of its characteristics in the revised manuscript.

We have made following changes in our manuscript that you will find at following line numbers in the revised manuscript track change version.

Line 310-313: “other occupations include casual wage labour outside tea estates, rickshaw pulling, firewood trading, transport-related work, and similar informal income-generating activities (coded as 4).”

Line 551-553 : “(occupations mainly consisted of casual and informal labour activities characterized by unstable earnings and limited access to employment-related benefits.)”

10. Are there any tea workers who have tertiary education and still work in tea garden and remain in poverty?

Response to comments 10:

“In response to this comment we have added following line that you will find at the line numbers in the revised manuscript track change version.

Line 441-445 : “Yet, tertiary education was found relatively uncommon (2%) in the study area suggesting that a small number of individuals with higher education stayed within tea-estate probably due to of family ties, limited employment opportunities, or engagement in supervisory and administrative activities related with tea-estate operations.”

11. Why income does not have significant implication in determining energy poverty should be revisited, because improved housing, number of earning members, occupations are closely linked with income. This finding may give a wrong message to the policymakers and tea garden owners regarding the long, undisputed and unfair low wages in the tea gardens of Bangladesh.

Response to comments 11:

“In response to this comment we have added following line that you will find at the line numbers 607-615 in the revised manuscript track change version.

Line 607-615: “However, the statistical insignificance of income should not be interpreted as evidence that household earnings are unimportant for reducing energy poverty. Instead, the impact of income may be indirectly captured by correlated socioeconomic factors such as housing quality, land ownership, occupational status, and the number of earning members, all of which represents underlying household welfare. This indicates that income does not exert an additional independent effect once these correlated characteristics are controlled for in the model. Nevertheless, income-generating opportunities and wage improvements remain central to addressing energy deprivation among tea workers. These results also”

12. This study underlines the importance of improving socioeconomic conditions of tea workers to help decrease their energy poverty but does not reflect on the issues of other practical dimensions of tea workers’ poverty that are associated with their subjugation, bonded labor and ghetto situation.

Response to comments 12:

“In response to this comment we have added following line that you will find at the line numbers 685-694 in the revised manuscript track change ver

Attachment Submitted filename: Response letter 4th Reviewer 10.06.2026.docx

# Editor report: Decision Letter 3

**DOI:** 10.1371/journal.pone.0354309.r007

**Authors:** Nitish Gogoi

6 Jul 2026

Household-level multidimensional energy poverty and its determinants: Evidence from tea estates in Bangladesh

PONE-D-25-43666R3

Dear Dr. Farid,

We’re pleased to inform you that your manuscript has been judged scientifically suitable for publication and will be formally accepted for publication once it meets all outstanding technical requirements.

Within one week, you’ll receive an e-mail detailing the required amendments. When these have been addressed, you’ll receive a formal acceptance letter, and your manuscript will be scheduled for publication.

An invoice will be generated when your article is formally accepted. Please note, if your institution has a publishing partnership with PLOS and your article meets the relevant criteria, all or part of your publication costs will be covered. Please make sure your user information is up-to-date by logging into Editorial Manager at Editorial Manager® and clicking the ‘Update My Information' link at the top of the page. For questions related to billing, please contact billing support.

If your institution or institutions have a press office, please notify them about your upcoming paper to help maximize its impact. If they’ll be preparing press materials, please inform our press team as soon as possible—no later than 48 hours after receiving the formal acceptance. Your manuscript will remain under strict press embargo until 2 pm Eastern Time on the date of publication. For more information, please contact onepress@plos.org.

Kind regards,

Nitish Gogoi, Ph.D.

Academic Editor

PLOS One

Additional Editor Comments (optional):

Reviewers' comments:
